# Tumor-specific surface marker–independent targeting of tumors through nanotechnology and bioorthogonal glycochemistry

**DOI:** 10.1172/JCI184964

**Published:** 2025-03-11

**Authors:** Hyesun Hyun, Bo Sun, Mostafa Yazdimamaghani, Albert Wielgus, Yue Wang, Stephanie Ann Montgomery, Tian Zhang, Jianjun Cheng, Jonathan S. Serody, Andrew Z. Wang

**Affiliations:** 1 Lineberger Comprehensive Cancer Center, University of North Carolina at Chapel Hill (UNC), Chapel Hill, North Carolina, USA.; 2 College of Pharmacy, Skaggs Pharmaceutical Sciences Center, University of Arizona, Tucson, Arizona, USA.; 3 Department of Pharmacology,; 4 Department of Pathology and Laboratory Medicine, and; 5 Division of Comparative Medicine, UNC, Chapel Hill, North Carolina, USA.; 6 Department of Medicine and Simmons Comprehensive Cancer Center, University of Texas Southwestern Medical Center, Dallas, Texas, USA.; 7 School of Engineering, Westlake University; Hangzhou, Zhejiang, China.; 8 Department of Medicine and; 9 Department of Immunology and Microbiology, UNC, Chapel Hill, North Carolina, USA.; 10 Department of Radiation Oncology, University of Texas Southwestern Medical Center, Dallas, Texas, USA.

**Keywords:** Oncology, Therapeutics, Cancer immunotherapy, Drug therapy, Nanotechnology

## Abstract

Biological targeting is crucial for effective cancer treatment with reduced toxicity but is limited by the availability of tumor surface markers. To overcome this, we developed a nanoparticle-based (NP-based), tumor-specific surface marker–independent (TRACER) targeting approach. Utilizing the unique biodistribution properties of NPs, we encapsulated Ac_4_ManNAz (Maz) to selectively label tumors with azide-reactive groups. Surprisingly, while NP-delivered Maz was cleared by the liver, it did not label macrophages, potentially reducing off-target effects. To exploit this tumor-specific labeling, we functionalized anti–4-1BB Abs with dibenzocyclooctyne to target azide-labeled tumor cells and activate the immune response. In syngeneic B16F10 melanoma and orthotopic 4T1 breast cancer models, TRACER enhanced the therapeutic efficacy of anti–4-1BB, increasing the median survival time. Immunofluorescence analyses revealed increased tumor infiltration of CD8^+^ T and NK cells with TRACER. Importantly, TRACER reduced the hepatotoxicity associated with anti–4-1BB, resulting in normal serum ALT and AST levels and decreased CD8^+^ T cell infiltration into the liver. Quantitative analysis confirmed a 4.5-fold higher tumor-to-liver ratio of anti–4-1BB accumulation with TRACER compared with conventional anti–4-1BB Abs. Our work provides a promising approach for developing targeted cancer therapies that circumvent limitations imposed by the paucity of tumor-specific markers, potentially improving efficacy and reducing off-target effects to overcome the liver toxicity associated with anti–4-1BB.

## Abstract 2

Development of a surface-biomarker independent strategy for cancer targeting

## Graphical abstract

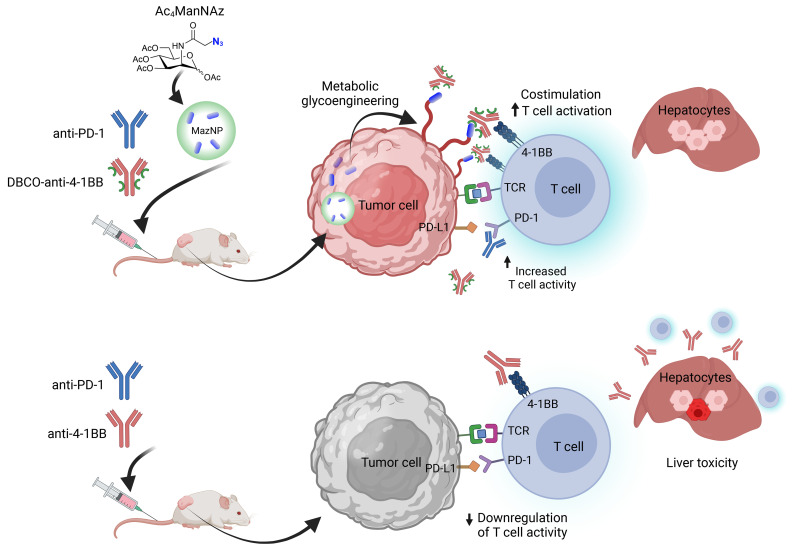

## Introduction

Tumor-specific targeting is critical to enhance antitumor efficacy while limiting normal tissue toxicity. In addition to agents that target cancer-specific signaling pathways, therapeutics — mostly mAbs — that can target tumor-specific antigens are clinically effective (1–6). Recently, there has been increased interest in the use of Ab-drug conjugates. These are Abs that bind to tumor surface markers and are coupled to cytotoxic compounds, which are released inside the tumor cell. Examples include trastuzumab deruxtecan, which targets human epidermal growth factor receptor type 2 (HER-2) in breast cancer, and lutetium-177-PSMA-617 for prostate cancer (7, 8).

Recent studies in nanoparticle-based (NP-based) cancer therapies have demonstrated promising results by effectively targeting tumor markers and enhancing therapeutic efficacy. Immunotherapies using NPs to target programmed death–ligand 1 (PD-L1) and polo-like kinase 1 (PLK1) have shown the potential of multitargeted approaches using markers overexpressed by tumor cells in lung cancer treatment (9). Additionally, anti–PD-L1 peptide–conjugated prodrug NPs, which combine PD-L1 blockade with immunogenic cell death, have exhibited efficient tumor targeting and robust antitumor immune responses in breast cancer models (10). Moreover, by conjugating anti-CD47 and anti–PD-L1 Abs onto the surfaces of NPs, Ab-conjugated, drug-loaded nanotherapeutics have enhanced antitumor efficacy. This approach facilitates targeted drug delivery directly to tumor tissues, leading to improved treatment outcomes in aggressive lung cancer models compared with conventional PD-L1 inhibitors (11).

While these approaches are promising for future translation, they rely on the presence of known tumor markers to target NPs. Thus, they do not circumvent a critical limitation of antitumor therapy, which is the need for the expression of a universal tumor-specific marker that can be targeted therapeutically (12–15). The lack of a universal tumor antigen hinders therapeutic immunotherapy by requiring a granular understanding of tumor-specific expression of proteins such as PD-L1 for NP targeting.

To address the limitation of targetable tumor-specific surface markers, our approach involves engineering targeting moieties on tumor cells through metabolic glycoengineering. This process integrates chemically reactive groups onto tumor cell surfaces (16), creating tumor-specific targets for therapeutic agents. Tumors exhibit a uniquely high metabolism (17), which can be leveraged in metabolic glycoengineering. For example, tetra-acetylated *N*-azidoacetyl-d-mannosamine (Ac_4_ManNAz; hereafter referred to as Maz), an analog of the common sialic acid derivative *N*-acetylneuraminic acid (Neu5Ac), can be utilized to label cancer cells in a dose-dependent manner with an enriched azido functional group (N_3_) (16). While Maz can be used to engineer abundant functional groups and surface markers on cell surfaces, its nonspecific labeling poses a challenge for cancer targeting, as it labels both cancer and normal cells (18–20). To overcome this, we used NP delivery of Maz to selectively target tumors and the tumor microenvironment (TME). We theorized that Maz would be degraded by macrophages in the liver, thus eliminating the potential toxicity associated with high liver uptake, a key shortcoming of NP biodistribution. Consequently, this strategy should selectively label tumors with reactive azide groups without extensive labeling of normal tissue.

Further enhancing this approach, bioorthogonal chemistry, particularly strain-promoted azide-alkyne cycloaddition (SPAAC), facilitates the specific binding of dibenzocyclooctyne (DBCO) to these azide-modified tumor cells, enabling rapid and specific in vivo reactions (20–22). Here, we implemented a NP-based, tumor-specific surface marker–independent approach via biorthogonal glycochemistry, termed TRACER. TRACER specifically refers to the utilization of DBCO-functionalized anti–4-1BB with Maz-encapsulated NPs (MazNPs). To assess this approach, we selected anti–4-1BB as a model therapeutic agent, as engagement of 4-1BB by its ligand enhances the T cell receptor (TCR) response to peptide and MHC. This leads to costimulatory signaling, which results in enhanced T cell expansion, effector function, resistance to apoptosis, and cytokine production (23–25). However, the clinical use of anti–4-1BB has been hampered by dose-limiting hepatotoxicity and systemic cytokine release syndrome (26). We posited that functionalizing anti–4-1BB with DBCO (DBCO–anti–4-1BB) would lead to selective targeting of tumor cells and circumvent the targeting of nonmalignant cells, thus substantially reducing its toxicity.

Here, we demonstrate that our TRACER approach, combining DBCO–anti–4-1BB with Maz-loaded NP (MazNP), selectively labeled tumor cell surfaces with azide groups and enhanced tumor accumulation of the DBCO-Ab conjugate. This strategy not only improved the efficacy of anti–4-1BB but also reduced dose-limiting hepatotoxicity. Our approach advances tumor targeting by utilizing nanotechnology and bioorthogonal glycochemistry, providing a method to enhance therapeutic specificity through targeted delivery of immunotherapeutic agents by selective labeling of tumor cells. This application of our approach repurposes the activity of anti–4-1BB to address the clinical challenge of hepatotoxicity associated with conventional anti–4-1BB therapies. Our TRACER approach selectively activated CD8^+^ T cells within the TME without developing off-target effects on nonmalignant hepatocytes. This selective targeting could considerably improve the clinical applicability of anti–4-1BB therapies, potentially reducing known hepatotoxic effects while enhancing therapeutic efficacy.

## Results

### Engineering of NP-encapsulating Maz and DBCO-functionalized anti–4-1BB.

**Figure 1:**
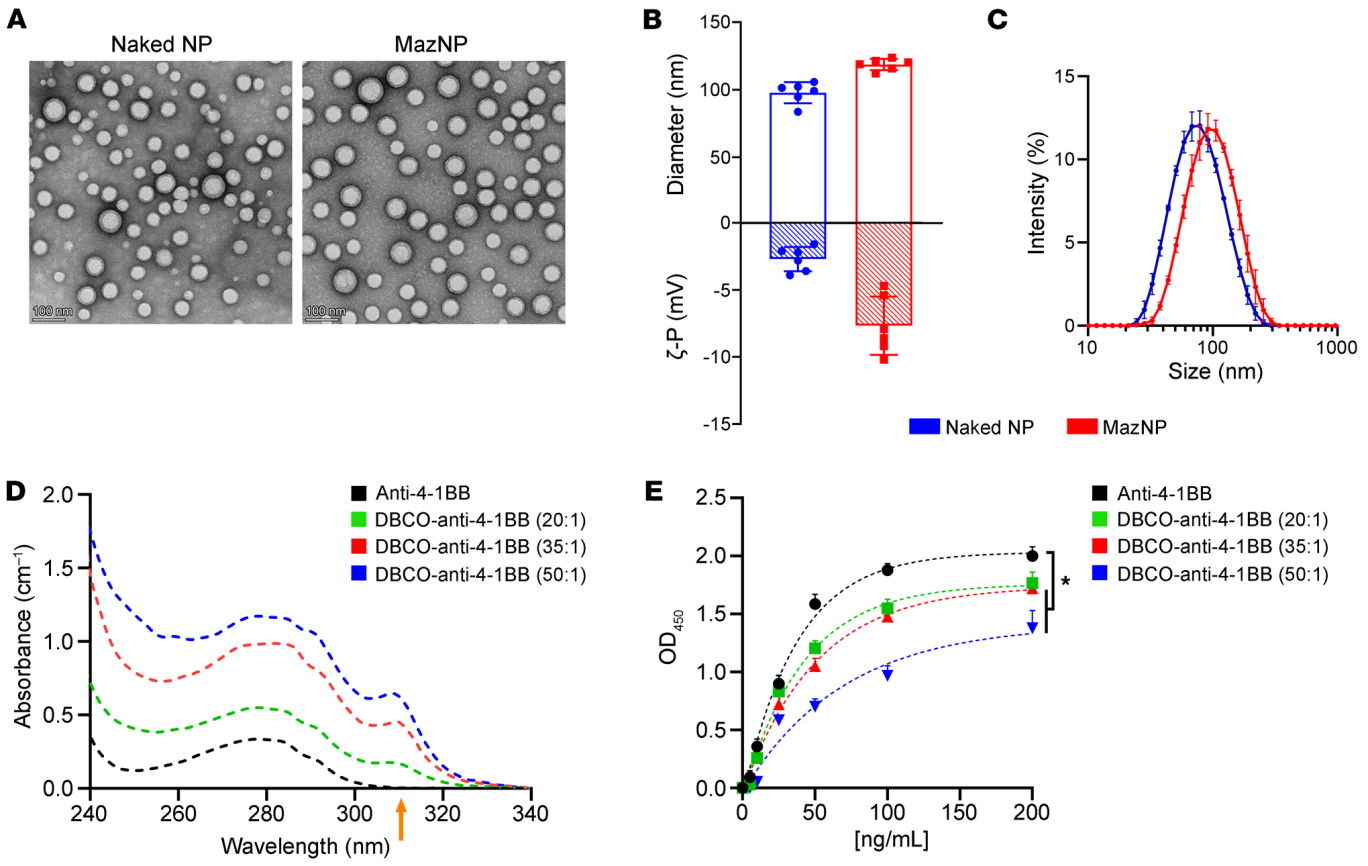
Engineering Abs and NPs for TRACER targeting delivery. (**A**) TEM images of naked NPs and MazNPs, negatively stained with 2% uranyl acetate. Scale bars: 100 nm. (**B**) Particle size and zeta potential (*n* = 6). (**C**) Intensity-based size distribution measured by DLS (*n* = 3). Samples were prepared identically and independently (mean ± SD). (**D**) UV spectra of unmodified anti–4-1BB and DBCO-functionalized anti–4-1BB with target molar ratios of conjugation of DBCO to anti–4-1BB (20:1, 35:1, and 50:1). The UV absorption band at 310 nm corresponds to absorbance from the conjugated DBCO group (arrow). (**E**) DBCO–anti–4-1BB binding affinity to 4-1BB protein, as determined by ELISA. *n* = 3 identically and independently prepared samples (mean ± SD). *P < 0.05 versus anti–4-1BB at 200 ng/mL, by Dunnett’s multiple-comparison test following 2-way ANOVA.

To specifically label tumors with reactive azide groups, Maz was used to generate azide functional groups on cell-surface glycans. These azide groups can conjugate with DBCO-functionalized biomolecules via in vivo bioorthogonal click reactions (18, 20). To enable this, we encapsulated Maz within methoxy poly(ethylene glycol)-b-poly(d,l-lactic-co-glycolic) acid (mPEG-PLGA) NPs using nanoprecipitation. The particle size, polydispersity index (PDI), and zeta potential of NPs are summarized in Supplemental Tables 1 and 2 (supplemental material available online with this article; https://doi.org/10.1172/JCI184964DS1). The PDI values of the NPs ranged from 0.11 to 0.21, indicating monodispersity. The average diameter, measured by dynamic light scattering (DLS), was 98 ± 8 nm for naked NPs and 119 ± 4 nm for MazNPs (Figure 1, B and C, and Supplemental Figure 1). Particle sizes, as measured by transmission electron microscopy (TEM), ranged from 50 to 80 nm (Figure 1A), and the Maz loading efficiency of MazNP was 6.3% ± 0.8% (Supplemental Figure 2). We also assessed potential aggregation and found that the size of MazNP in 50% serum (129.6 ± 1.0 nm; PDI: 0.23 ± 0.03, at 10 mg/mL) was similar to that measured in 10 mM NaCl (122.9 ± 7.2 nm; PDI: 0.20 ± 0.02) (Supplemental Table 3). These data demonstrate in vivo stability and lack of aggregation for the NPs.

To enable tumor-targeted delivery of anti–4-1BB, we formulated a DBCO-functionalized anti–4-1BB mAb (DBCO–anti–4-1BB) that could react with the azide group on Maz. DBCO–anti–4-1BB was synthesized by coupling the NHS-ester–modified DBCO ligand with the primary amines on the anti–4-1BB mAb. The target DBCO/anti–4-1BB molar ratios were 20:1, 35:1, and 50:1 based on our previous work (27). The actual degrees of functionalization (DOF) of anti–4-1BB with DBCO were 8, 16, and 23, respectively, as determined by ultraviolet-visible (UV) spectroscopy (Figure 1D). Conjugation was further confirmed using matrix-assisted laser desorption ionization time-of-flight mass spectroscopy (MALDI-TOF MS) (Supplemental Figure 3A). An increase in the mass of anti–4-1BB was observed post-reaction, indicating the addition of DBCO-reactive groups. The DOFs determined using the MALDI-TOF MS method were higher than those determined by the UV spectroscopic method, since DBCO-PEG_13_-NHS contains only 90 mol% of the DBCO moiety (Supplemental Figure 3B).

To determine whether DBCO conjugation affects Ab binding, we evaluated the binding of the murine 4-1BB ligand with different concentrations of unmodified or modified anti–4-1BB using ELISA (Figure 1E). Using a ratio of 20:1, DBCO–anti–4-1BB retained its binding to 4-1BB. However, we noted a marked reduction in the binding affinity of DBCO–anti–4-1BB at the target DOFs of 35:1 and 50:1. This indicated that a high degree of Ab modification with DBCO could compromise Ab binding to the DBCO ligand, due to steric hindrance caused by the bulky DBCO moiety (27). To take advantage of DBCO conjugation to the Ab without compromising binding, we selected DBCO–anti–4-1BB with the target DOF of 20:1 for further studies.

### TRACER improves therapeutic efficacy.

**Figure 2:**
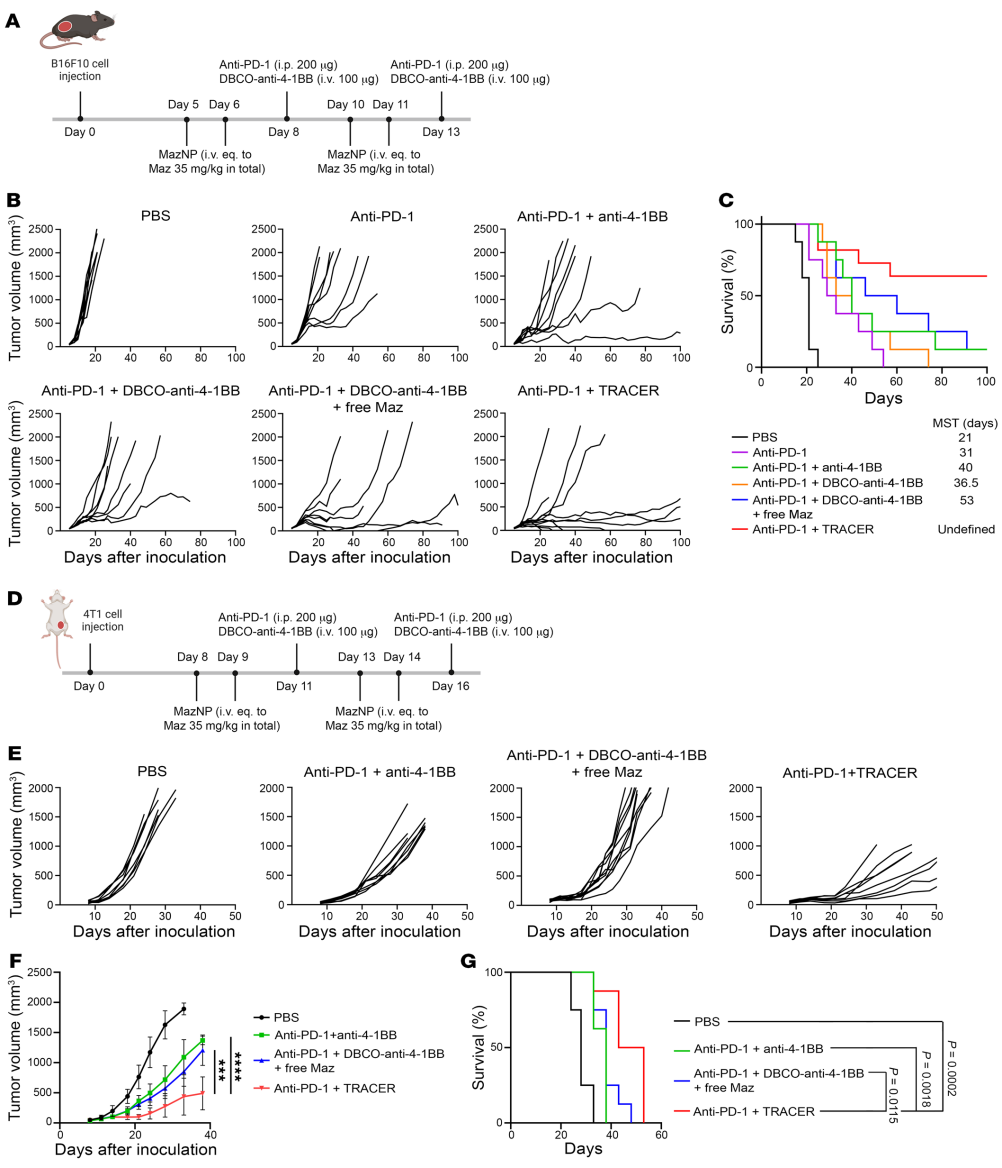
TRACER improves the efficacy of anti–4-1BB in vivo. (**A**) Dosing schedule of Abs and NPs for B16F10 tumor–bearing mice. (**B**) Individual tumor growth curves of B16F10 tumors in C57BL/6 mice treated with PBS, anti–PD-1, anti–PD-1+anti–4-1BB, anti–PD-1+DBCO–anti–4-1BB, anti–PD-1 plus DBCO–anti–4-1BB plus free Maz, or anti–PD-1 plus TRACER (*n* = 8–11 per group). (**C**) Kaplan-Meier survival curves for B16F10 tumor–bearing mice. (**D**) Dosing schedule of treatments in orthotopic 4T1 breast tumor–bearing mice. (**E**) Individual tumor growth curves of 4T1 breast tumors in BALB/c mice treated with PBS, anti–PD-1 plus anti–4-1BB, anti–PD-1 plus DBCO–anti–4-1BB plus free Maz, or anti–PD-1 plus TRACER (*n* = 8 per group. (**F**) Average tumor growth curves for animals shown in **E** (mean ± SD). Tumor growth over time was compared by Šidák’s multiple-comparison test following 2-way ANOVA; ***P < 0.001 and ****P < 0.0001. (**G**) Kaplan-Meier survival curves of 4T1 tumor–bearing mice. *P* values were calculated using the log-rank test.

We investigated whether DBCO–anti–4-1BB with MazNP (TRACER) enhances the efficacy of immunotherapy in the poorly immunogenic B16F10 melanoma syngeneic tumor model, which responds poorly to checkpoint therapies such as single-agent treatment or combinations of anti–4-1BB and anti–CTLA-4 antibodies (28). C57BL/6 mice were given B16F10 tumors and then received either MazNP or free Maz i.v. Two days later, when the tumors reached an average size of approximately 120 mm^3^ (Figure 2A), the animals were treated with anti–4-1BB or DBCO–anti–4-1BB. All experimental groups also received anti–programmed death 1 (anti–PD-1) mAb treatment. We found that anti–PD-1 plus TRACER-targeted anti–4-1BB was highly effective, resulting in a longer median survival time (MST) (>100 days) compared with controls: anti–PD-1 plus free anti–4-1BB (MST = 40 days, *P* = 0.0071) and anti–PD-1 plus DBCO–anti–4-1BB (MST = 36.5 days, *P* = 0.0071) (Figure 2B and C). We observed improvement in survival among the mice that received TRACER compared with those that received anti–PD-1 plus DBCO–anti–4-1BB and free Maz, however, this change did not reach the predefined definition of statistical significance (*P* = 0.0697) (Figure 2C and Supplemental Table 4). This trend is reflected by the substantial difference in survival rates (63.6% MazNP vs. 12.5% free Maz) and MST (100 days vs. 53 days), demonstrating an improvement in survival outcomes in the MazNP-treated group. It is important to note that 4 of 11 mice in the experimental arm had no evidence of tumor for 100 days. These results indicate a potentially important therapeutic benefit that warrants further investigation.

To validate our in vivo results, we evaluated TRACER’s antitumor efficacy in the 4T1 tumor model, which responds poorly to single-agent checkpoint therapy (29). 4T1 cells were injected into a mammary fat pad, and tumor-bearing mice were treated with anti–PD-1 plus TRACER or the control therapy (Figure 2D). Similar to our findings in the B16F10 melanoma model, mice treated with anti–PD-1 plus TRACER showed improved tumor control and overall survival (Figure 2, E–G), compared with PBS treatment (MST = 28 days) and compared with anti–PD-1 plus anti–4-1BB (MST = 38 days) or anti–PD-1 plus DBCO–anti–4-1BB with free Maz (MST = 38 days) (Figure 2G). Our data show that the targeted delivery of anti–4-1BB via bioorthogonal glycochemistry increased therapeutic efficacy in 2 tumor models poorly responsive to checkpoint inhibitor therapy.

### Therapeutic efficacy of TRACER involves both innate and adaptive immunity.

**Figure 3:**
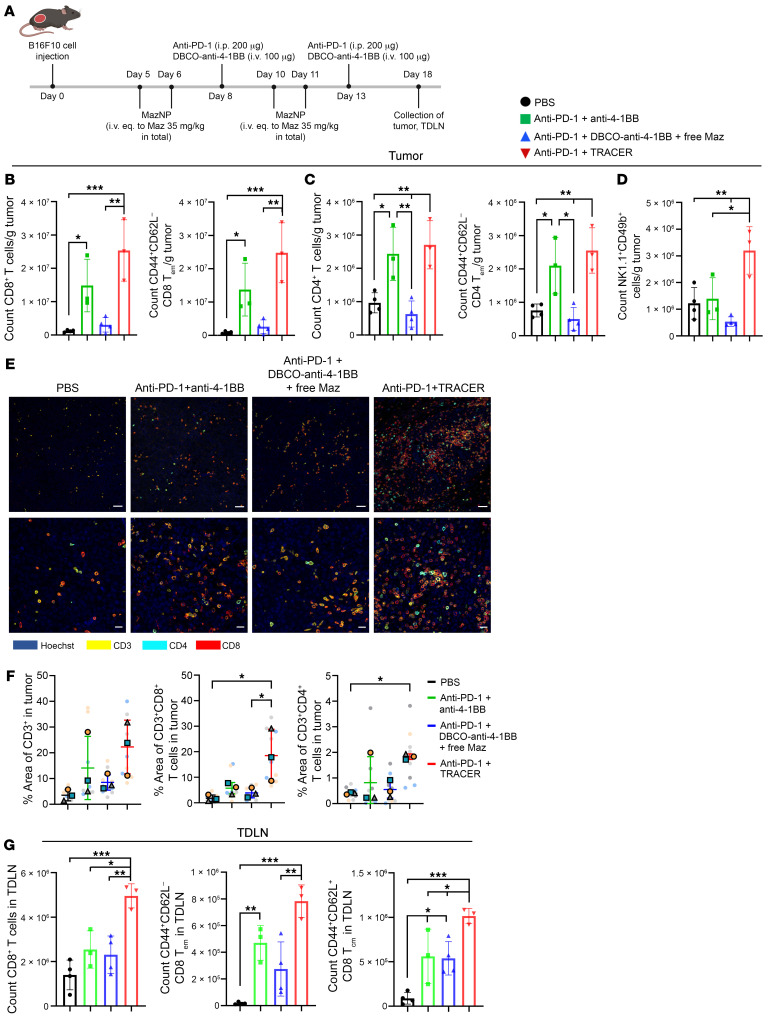
TRACER delivery of anti–4-1BB increases CD8^+^ T cell expansion and NK cell activation in a B16F10 melanoma model. (**A**) Schematic of B16F10 tumor inoculation, treatments, and time points for collection of tumors and TDLNs. (**B**) Quantitation of CD8^+^ T cells and CD44^+^CD62L^–^ Tem cells in CD8^+^ T cells, (**C**) CD4^+^ T cells or CD44^+^CD62L^–^ Tem cells in CD4^+^ T cells in tumors, or (**D**) NK1.1^+^CD49b^+^ cells in tumors (*n* = 3–4 independent animals; mean ± SD). *P < 0.05, **P < 0.01, and ***P < 0.001, by Tukey’s multiple-comparison test following 1-way ANOVA. (**E**) Representative IF images of B16F10 tumor sections (shown again in Supplemental Figure 8). Scale bars: 50 μm (first row) and 20 μm (second row). (**F**) Quantitative analysis of IF staining of tumor sections. The percentage of area of CD3^+^ T cells was estimated as the area of CD3^+^ fluorescence (yellow), divided by the tissue in the field of view area (outlined by blue Hoechst 33258 staining). The percentage of area of CD3^+^CD8^+^ or CD3^+^CD4^+^ T cells was calculated as the area of CD8^+^ (red) or CD4^+^ (cyan) fluorescence overlapped with CD3^+^ (yellow), divided by the area of the tissue in the field of view (outlined by blue Hoechst 33258 staining). Randomly selected fields (9 images per group; Supplemental Figure 8) were analyzed with Fiji software. For statistical analysis, image data from 3 fields per mouse were averaged to generate a single value for each biological replicate (*n* = 3 mice per group). Each biological replicate is color coded: gray triangles, blue squares, and orange dots represent individual animals. *P < 0.05, by Tukey’s multiple-comparison test following 1-way ANOVA. (**G**) Enumeration of CD8^+^ T cells, CD44^+^CD62L^–^ Tem cells, and CD44^+^CD62L^+^ Tcm cells from CD8^+^ T cells in TDLNs (*n* = 3–4 independent animals, mean ± SD). *P < 0.05, **P < 0.01, and ***P < 0.001, by Tukey’s multiple-comparison test following 1-way ANOVA. All data are shown as the mean ± SD; each symbol represents 1 individual mouse.

Next, we evaluated the mechanisms underlying the enhanced antitumor activity of TRACER. For this evaluation, adaptive and innate immune cells in the TME and tumor-draining lymph nodes (TDLNs) in the B16F10 melanoma model were assessed by flow cytometry (Figure 3A and Supplemental Figure 4). Mice receiving anti–PD-1 plus TRACER showed an increase in CD8^+^ T cells compared with those treated with PBS or anti–PD-1 plus DBCO–anti–4-1BB with free Maz (Figure 3B and Supplemental Figure 5). Although statistical analysis did not reveal a significant difference between the anti–PD-1 plus TRACER and the anti–PD-1 plus anti–4-1BB treatments, the data showed a trend toward increased CD8^+^ T cells in the TRACER group. Notably, the TRACER approach also led to expanded effector memory CD8^+^ T (Tem) cells (CD44^+^CD62L^–^) compared with the PBS control and anti–PD-1 plus DBCO–anti–4-1BB plus free Maz, suggesting a potential shift toward a memory-oriented cytotoxic phenotype within the TME (Figure 3B and Supplemental Figure 6A). Conversely, we noted an increase in CD4^+^ T cells and Tem cells in the TRACER group (Figure 3C, Supplemental Figure 5, and Supplemental Figure 6B). However, this increase was less pronounced compared with that observed in the CD8^+^ T cells, indicating that the antitumor effects of the TRACER approach predominantly enhanced the quantitation of CD8^+^ T cells in the tumor. Additionally, we observed an increase in the frequency and quantitation of NK cells (NK1.1^+^CD49^+^) in the TRACER group compared with the groups receiving nontargeted anti–4-1BB treatments, including anti–PD-1 plus anti–4-1BB and anti–PD-1 plus DBCO–anti–4-1BB with free Maz (Figure 3D and Supplemental Figure 7). Collectively, these results suggest that TRACER effectively enhanced the recruitment and expansion of both NK and CD8^+^ T cells in the tumor, which correlated with an improved antitumor immune response.

Immunofluorescence (IF) staining was used to identify CD3^+^, CD4^+^, and CD8^+^ T cells within the TME, revealing the spatial localization of immune cells (Figure 3E and Supplemental Figure 8). Confocal microscopy showed enhanced CD3^+^ T cell infiltration into the tumors of mice treated with anti–PD-1 plus TRACER when compared with those treated with PBS control and anti–PD-1 plus DBCO–anti–4-1BB with free Maz. Quantitative analysis revealed that the percentage of CD3^+^CD8^+^ T cells within tumors treated with anti–PD-1 plus TRACER (18.5% ± 10.3% of tissue area in the field of view) was significantly higher than tumors treated with PBS (1.8% ± 1.4%) or anti–PD-1 plus DBCO–anti–4-1BB with free Maz (3.9% ± 2.4%), whereas neither anti–PD-1 plus anti–4-1BB nor anti–PD-1 plus DBCO–anti–4-1BB with free Maz induced tumor infiltration of CD3^+^CD8^+^ T cells when compared with the PBS control group (Figure 3F). Furthermore, 4-1BB signaling also stimulated CD4^+^ effector T cells to expand and produce proinflammatory cytokines, such as IFN-γ and TNF-α, providing a proinflammatory environment that favored tumor rejection (30, 31). There was a higher percentage of CD3^+^CD4^+^ T cells in tumors treated with anti–PD-1 plus TRACER (1.8% ± 1.0%) than in those of mice treated with PBS (0.4% ± 0.2%). In TDLNs, the anti–PD-1 plus TRACER not only increased the number of CD8^+^ T cells and central memory T cells (Tcm) cells CD44^+^CD62L^+^), but also elevated numbers of CD8^+^ Tem cells compared with the controls and nontargeted anti–4-1BB groups (Figure 3G and Supplemental Figure 9). Polymorphonuclear myeloid-derived suppressor cells (PMN-MDSCs) in TDLNs promote cancer progression and are associated with a poor prognosis (32, 33). Increased infiltration of PMN-MDSCs (CD11b^+^Ly6C^lo^Ly6G^+^) in TDLNs was observed in all groups, except for those treated with anti–PD-1 plus TRACER or PBS control (Supplemental Figure 10). This suggests that TRACER, facilitated by MazNP, did not lead to PMN-MDSC accumulation in TDLNs, probably due to its enhanced specificity in delivering anti–4-1BB to the tumor site.

**Figure 4:**
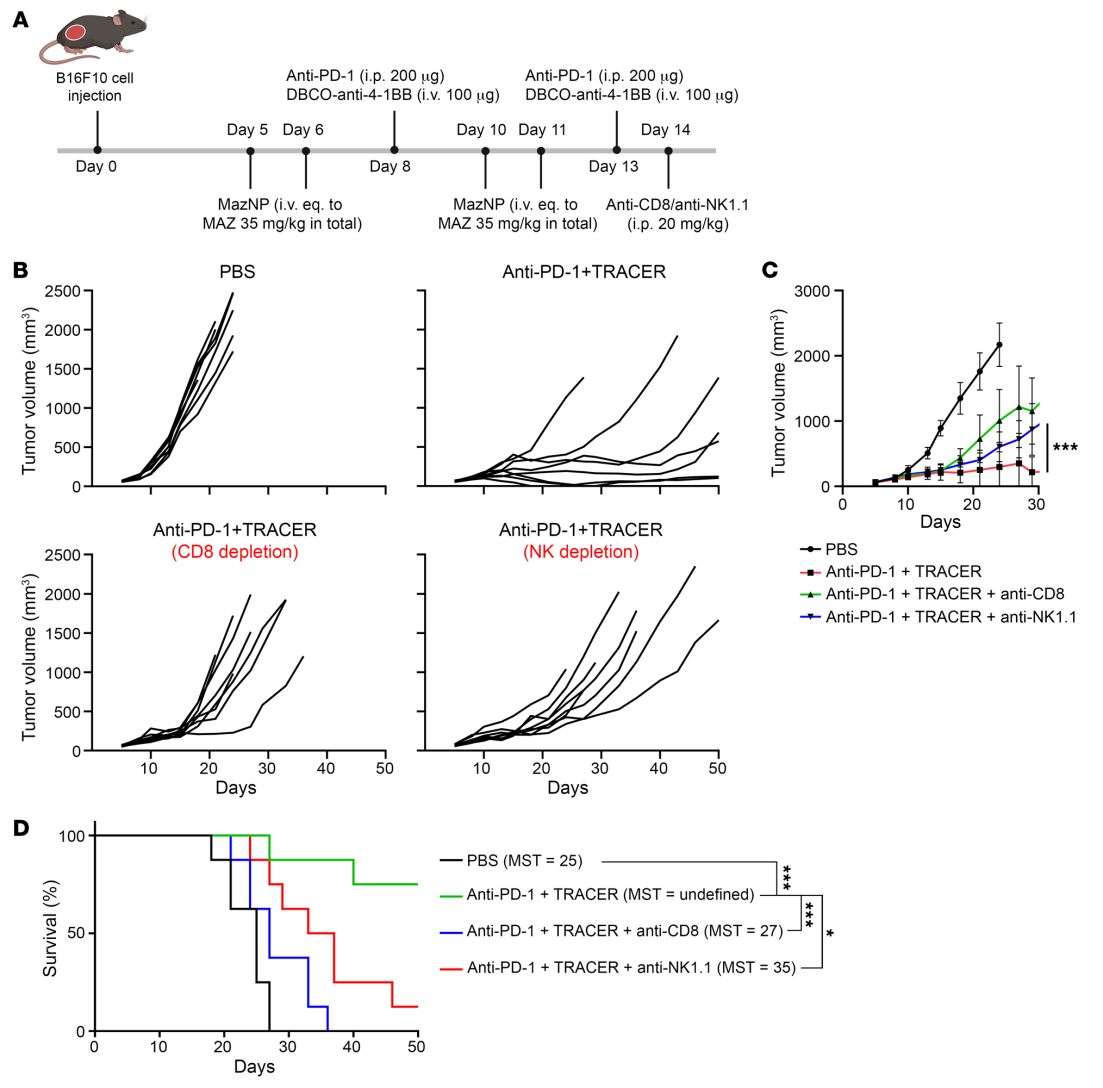
The antitumor efficacy of TRACER targeting is abrogated by the depletion of CD8^+^ T cells or NK cells in the B16F10 melanoma model. (**A**) Schematic of B16F10 tumor inoculation, treatments, and the depletion of CD8^+^ T cells or NK cells. (**B**) Individual growth curves of B16F10 tumors in animals treated with anti–PD-1 plus TRACER with or without CD8^+^ T cell or NK cell depletion (*n* = 8 per group). (**C**) Average tumor growth curves for each treatment shown in **B**. ***P < 0.001, by Šidák’s multiple-comparison test following 2-way ANOVA. (**D**) Differences in survival were determined for each group using the Kaplan-Meier method. *P < 0.05 and ***P < 0.001, by log-rank test.

To verify the role of immune cells in the robust antitumor efficacy of anti–PD-1 plus TRACER, we depleted CD8^+^ T cells or NK cells in mice bearing a B16F10 tumor on day 14, one day after the final treatment, and monitored tumor growth for 50 days (Figure 4A). The effects of TRACER on tumor regression (Figure 4, B and C) and overall survival were lost after CD8^+^ T cell depletion (Figure 4D). Mice treated with anti–PD-1 plus TRACER, followed by CD8^+^ T cell depletion, had a MST of 27 days. This was significantly shorter than that for mice treated with anti–PD-1 plus TRACER (MST >50 days; *P* < 0.0005 vs. CD8^+^ depletion) and was comparable to that for PBS-treated animals (MST = 25 days). Similarly, depletion of NK cells in mice with B16F10 tumors substantially reduced the efficacy of anti–PD-1 plus TRACER treatment. Thus, these data show that the antitumor activity of TRACER was dependent on the function of CD8^+^ and NK cells.

### TRACER reduces hepatotoxicity of anti–4-1BB.

**Figure 5:**
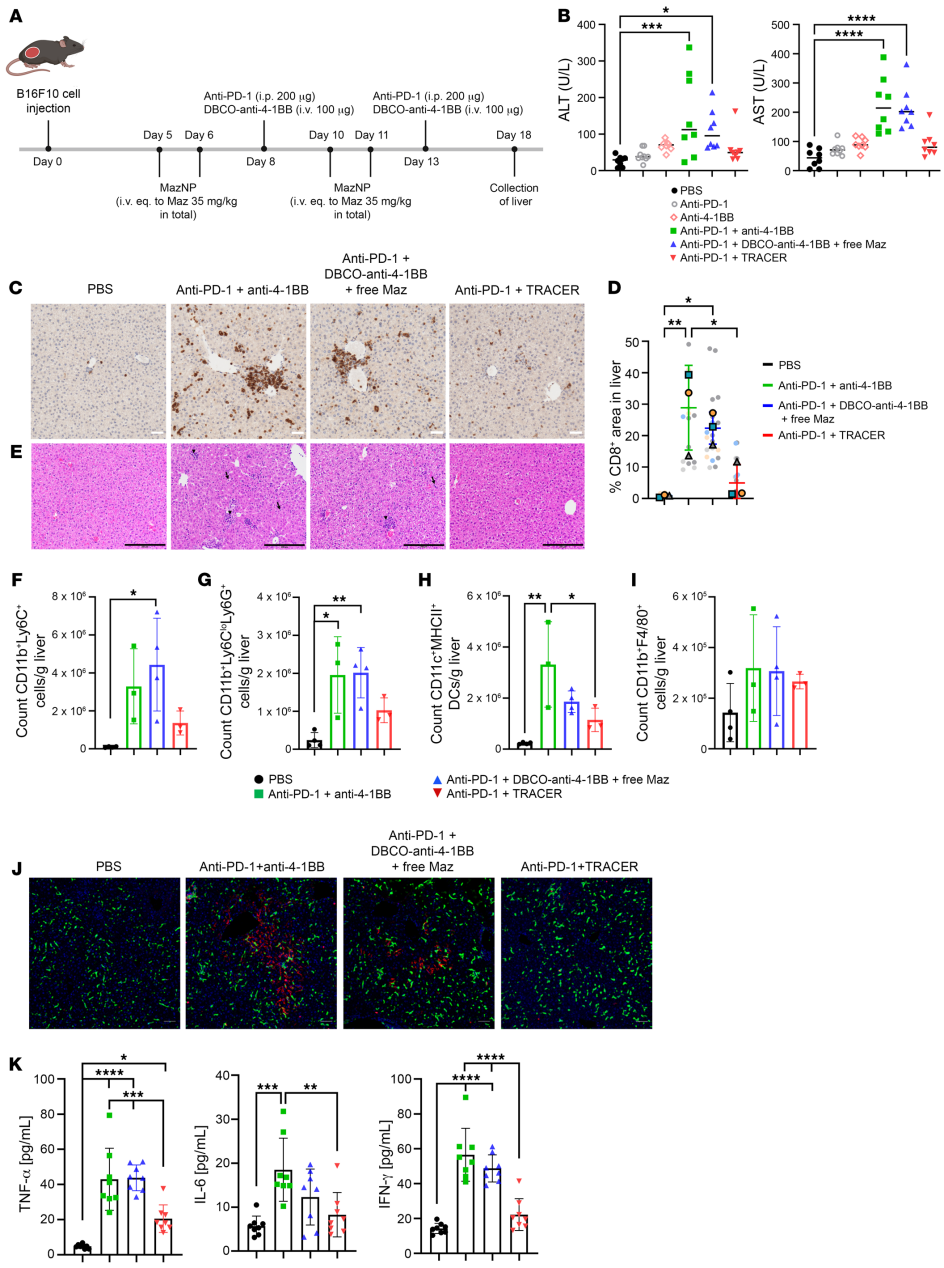
MazNP does not induce anti–4-1BB liver toxicity in B16F10 tumor–bearing mice. (**A**) Schematic of B16F10 tumor inoculation, treatments, and time points for liver collection. (**B**) Serum levels of ALT and AST measured as units of enzyme per liter (U/L) (*n* = 8 per group). *P < 0.05, ***P < 0.001, and ****P < 0.0001 versus PBS, by Dunnett’s multiple-comparison test following 1-way ANOVA. (**C**) IHC staining for CD8^+^ on sectioned liver tissues (shown again in Supplemental Figure 12). Scale bars: 50 μm. (**D**) Quantification of CD8^+^ T cells infiltrated into the liver in mice (see IHC images in Supplemental Figure 12). The percentage of CD8^+^ T cell area in the liver was estimated as the area of CD8^+^ (stained brown with DAB) in the field of view (outlined by hematoxylin counterstaining). Randomly selected fields were analyzed using Fiji software: 11 images for PBS; 17 images for anti–PD-1 plus anti–4-1BB; 20 images for anti–PD-1 plus DBCO–anti–4-1BB plus free Maz; and 21 images for anti–PD-1 plus TRACER. For statistical analysis, image data from multiple fields per mouse were averaged to generate a single value for each biological replicate (*n* = 3 mice per group). Each biological replicate is colored coded: gray triangles, blue squares, and orange dots represent individual animals. *P < 0.05 and **P < 0.01, by Tukey’s multiple-comparison test following 1-way ANOVA. (**E**) H&E staining of representative liver tissue slides. Scale bars: 200 μm. Counts of (**F**) M-MDSCs (CD11b^+^Ly6C^+^), (**G**) PMN-MDSCs (CD11b^+^Ly6C^lo^Ly6G^+^), (**H**) DCs (CD11c^+^MHCII^+^), and (**I**) macrophages (CD11b^+^F4/80^+^) in livers (*n* = 3–4 independent animals, mean ± SD). *P < 0.05 and **P < 0.01, by Tukey’s multiple-comparison test following 1-way ANOVA. (**J**) IF images of activated liver-resident CD163^+^ (green) and/or CD206^+^ (red) cells. Scale bars: 50 μm. (**K**) Serum levels of TNF-α, IL-6, and IFN-γ measured by ELISA (*n* = 8 per group, mean ± SD). *P < 0.05, **P < 0.01, ***P < 0.001, and ****P < 0.0001, by Tukey’s multiple-comparison test following 1-way ANOVA. All data are shown as the mean ± SD; each symbol represents 1 individual mouse.

The use of agonistic Abs targeting 4-1BB has been hampered by systemic and hepatic toxicity, which has considerably decreased enthusiasm for this approach. Given the increased efficacy of TRACER, we were interested in evaluating whether this treatment increases toxicity. Considering the uptake of NPs by macrophages, high levels of azide labeling in liver macrophages following NP uptake could lead to substantial hepatotoxicity. Using the same study design as for our therapeutic efficacy studies, we examined hepatotoxicity in mice on day 18, which was 10 days after initiating treatment (Figure 5A). Surprisingly, we found that TRACER did not increase hepatotoxicity, but instead reduced hepatotoxicity, despite the increased liver uptake of NPs. As shown in Supplemental Figure 11, we observed weight-based enlargement of the spleen and liver in both anti–PD-1 plus anti–4-1BB and anti–PD-1 plus DBCO–anti–4-1BB with free Maz treatment groups, and toxicity was consistent with the known hepatotoxicity profile of anti–4-1BB in mice (34). However, this was not observed in the anti–PD-1 plus TRACER group. Serum liver enzyme analysis further supported this finding, with significantly elevated levels of alanine transaminase (ALT) and aspartate aminotransferase (AST) in the groups receiving nontargeted anti–4-1BB, as previously reported (28, 35). In contrast, mice that received TRACER had normal serum ALT and AST levels (Figure 5B), demonstrating that delivery of anti–4-1BB using the TRACER process eliminated the hepatotoxicity found with anti–4-1BB treatment alone.

To investigate this mechanism, we characterized immune activation differences in the liver between the experimental groups using IHC to quantify liver-infiltrating CD8^+^ T cells (Figure 5C and Supplemental Figure 12). We found that both anti–PD-1 plus anti–4-1BB and anti–PD-1 plus DBCO–anti–4-1BB with free Maz significantly increased CD8^+^ T cell infiltration into the liver. Contrastingly, anti–PD-1 plus TRACER reduced CD8^+^ T cell accumulation. Image analysis showed increased CD8^+^ T cell accumulation in the anti–PD-1 plus anti–4-1BB group (29.8% ± 18.2% of tissue area in the field of view), which was significantly greater than the PBS control group (0.9% ± 0.6%) and anti–PD-1 plus TRACER (5.4% ± 5.8%) (Figure 5D). While the anti–PD-1 plus DBCO–anti–4-1BB with free Maz group (22.1% ± 10.9%) showed a trend toward higher CD8^+^ T cell accumulation compared with the TRACER group, this difference was not statistically significant. However, CD8^+^ T cell accumulation in the free Maz treatment group remained significantly higher than in the PBS control. Histologic and morphologic liver analysis further revealed that anti–PD-1 plus anti–4-1BB and anti–PD-1 plus DBCO–anti–4-1BB with free Maz increased immune cells surrounding portal triads and in sinusoids (Figure 5E), consistent with liver injury (Figure 5B). However, mice receiving TRACER did not show increased immune cell infiltration into the liver.

To confirm these findings, we performed flow cytometry to analyze immune cell infiltration into the liver (Figure 5, F–I). Compared with the PBS control group, the anti–PD-1 plus TRACER group had a less pronounced increase in monocytic myeloid-derived suppressor cells (M-MDSCs) (CD11b^+^Ly6C^+^) than both the anti–PD-1 plus anti–4-1BB and anti–PD-1 plus DBCO–anti–4-1BB with free Maz groups, which had increased numbers of M-MDSCs in the liver (Figure 5F and Supplemental Figure 13). Additionally, there was an increase in the number of PMN-MDSCs (CD11b^+^Ly6C^lo^Ly6G^+^) and DCs (CD11c^+^MHCII^+^) in the nontargeted anti–4-1BB groups, unlike in the TRACER group, which was similar to the PBS control (Figure 5, G and H, Supplemental Figure 13, and Supplemental Figure 14A). No notable differences were found in the number of liver macrophages (CD11b^+^F4/80^+^) between nontargeted anti–4-1BB and TRACER-delivered anti–4-1BB treatments (Figure 5I and Supplemental Figure 14B). IF staining for the macrophage activation markers CD163 and CD206 (36, 37) in liver sections revealed increased expression of CD206^+^ macrophages in both the anti–PD-1 plus anti–4-1BB and anti–PD-1 plus DBCO–anti–4-1BB with free Maz groups, indicative of liver injury and inflammation (36–38) (Figure 5J). Interestingly, the anti–PD-1 plus TRACER group did not show an increase in CD206^+^ macrophages, reflecting decreased liver inflammation in mice receiving TRACER, as CD206-expressing macrophages are elevated after tissue damage.

Next, we evaluated treatment effects on proinflammatory cytokine serum levels (Figure 5K). We observed increases in TNF-α levels in all groups compared with controls, with the greatest difference seen in mice that received anti–PD-1 plus anti–4-1BB or anti–PD-1 plus DBCO–anti–4-1BB with free Maz. No difference was found between PBS and TRACER groups in the concentrations of IL-6 or IFN-γ, which were significantly higher in the nontargeted anti–4-1BB groups (Figure 5K). These data indicate that TRACER administration was associated with reduced systemic inflammation compared with anti–PD-1 plus anti–4-1BB or DBCO–anti–4-1BB with free Maz.

### MazNP does not generate azide groups on macrophage surfaces.

**Figure 6:**
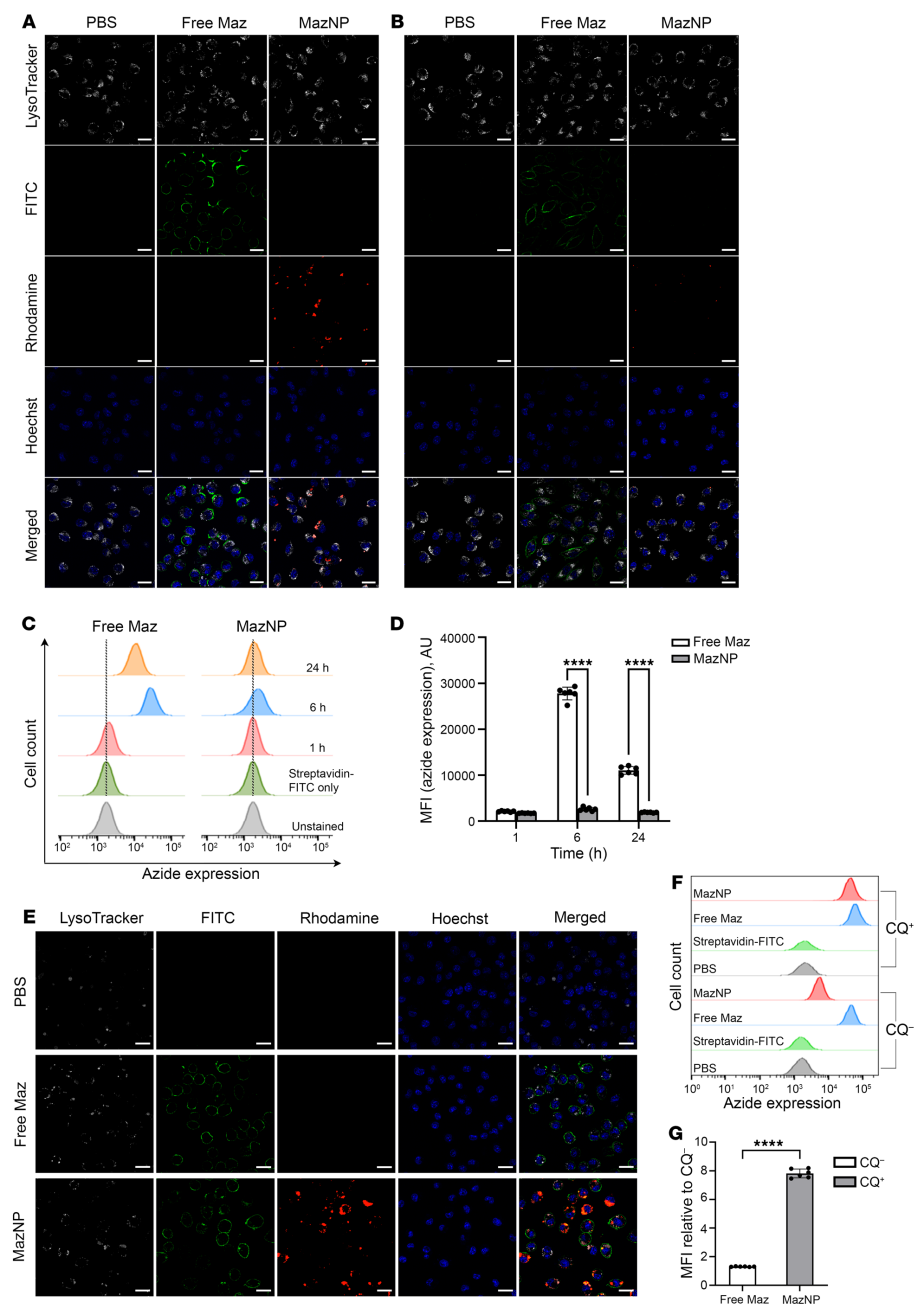
MazNP does not generate azide groups on macrophage surfaces. (**A**) Azide group generation on the surface of J774A.1 macrophages incubated with PBS, free Maz, or non-PEGylated MazNP for 6 hours or (**B**) 24 hours. Cells were imaged with a confocal microscope (white: LysoTracker; green: streptavidin-FITC; red: rhodamine-labeled MazNP; blue: nuclei stained with Hoechst 33258). Scale bars: 10 μm. (**C**) Time-dependent cell-surface azide expression following treatments with free Maz or MazNP, as determined by flow cytometry. The representative flow cytometry histogram shows cell-surface azide expression at 1 hour, 6 hours, and 24 hours after treatment. (**D**) Quantification of cell-surface azide expression, shown as the MFI of streptavidin-FITC, as presented in **C**. *n* = 6 identically and independently prepared samples (mean ± SD). ****P < 0.0001, by Šidák’s multiple-comparison test following 2-way ANOVA. (**E**) Azide group generation on J774A.1 macrophages treated with CQ prior to a 6-hour incubation with PBS, free Maz, or non-PEGylated MazNP. Cells were imaged with a confocal microscope (green: streptavidin-FITC; red: rhodamine-labeled MazNP; blue: nuclei stained with Hoechst 33342). Scale bars: 10 μm. (**F**) Representative flow cytometry histograms showing cell-surface azide expression with or without CQ pretreatment in the free Maz and MazNP groups. (**G**) Quantification of cell-surface azide expression shown as the ratio of MFI of streptavidin-FITC relative to CQ^–^(CQ^+^ to CQ^–^). *n* = 6 identically and independently prepared samples (mean ± SD). ****P < 0.0001, by unpaired, 2-tailed *t* test.

Addressing hepatotoxicity after anti–4-1BB treatment is crucial for clinical applicability. Despite the high liver distribution of NPs following systemic administration due to macrophage uptake (39, 40), we observed reduced liver inflammation and macrophage activation in mice receiving anti–PD-1 plus TRACER. To investigate mechanisms underlying this finding, we hypothesized that MazNP is uniquely metabolized in macrophages, thereby limiting the labeling of azides on these cells. To assess this, we compared the macrophage-labeling efficiency of MazNP with free Maz in vitro using non-PEGylated MazNPs. J774A.1 macrophages were incubated with either free Maz or rhodamine-labeled, non-PEGylated MazNP for 6 hours. Surface azide groups were detected using DBCO–PEG4-biotin and visualized with fluorescently labeled streptavidin (streptavidin-FITC). Confocal microscopy showed greater fluorescence intensity in the free Maz group, confirming the presence of azide on the macrophage surface (Figure 6A). This was not observed in macrophages incubated with MazNPs, where the NPs colocalized with LysoTracker, indicating their presence in lysosomes. Flow cytometric analysis further confirmed these findings, revealing significantly higher levels of cell-surface azide expression in the free Maz group compared with the MazNP group at both the 6-hour and 24-hour time points (Figure 6, C and D). We also analyzed the cell-labeling activity of MazNP in B16F10 cells (Supplemental Figure 15 and Supplemental Figure 17A). Interestingly, we found no difference in the labeling of surface azide groups in B16F10 cells when comparing the free Maz and MazNP groups. These data suggest that MazNPs are trafficked to lysosomes in macrophages, limiting their availability to label membrane azides.

To further investigate this hypothesis, we evaluated MazNP trafficking and accumulation in lysosomes at different time points using LysoTracker. MazNP colocalized with LysoTracker at 6 hours, but this colocalization was not observed at 24 hours (Figure 6B). Flow cytometry using rhodamine-labeled MazNP in macrophages showed initial uptake at 1 hour, with a peak in cellular uptake at 6 hours and a dramatic decrease at 24 hours, with levels falling below the initial uptake at 1 hour (Supplemental Figure 16). In contrast, B16F10 cells showed prolonged intracellular retention of MazNP, with signal intensity increasing from 1 hour to 6 hours and remaining persistent through 24 hours (Supplemental Figure 17B). These data suggest distinct intracellular processing of MazNP in macrophages compared with that in tumor cells.

To confirm the lysosomal trafficking of MazNP in macrophages, we evaluated the effects of chloroquine (CQ), a lysosomal inhibitor, on cell-surface azide expression. Confocal microscopy revealed that J774A.1 macrophages pretreated with CQ and treated with MazNP showed increased surface azide labeling (Figure 6E). Flow cytometric analysis quantified this increase, showing approximately 8-fold higher surface azide expression in CQ-pretreated cells with MazNP, whereas CQ pretreatment had no effect on azide labeling in macrophages exposed to free Maz (Figure 6, F and G). Additionally, flow cytometric analysis revealed a significantly higher rhodamine signal from MazNP in CQ-treated macrophages (Supplemental Figure 18), indicating enhanced intracellular accumulation when lysosomal degradation was inhibited. These data demonstrate that Maz encapsulated in NPs was trafficked to lysosomes for degradation, while free Maz directly labeled surface azides in macrophages. This provides a potential mechanism for the reduced toxicity observed in mice treated with TRACER plus anti–PD-1.

To further understand the role of lysosomal processing, we preincubated B16F10 cells with Earle’s balanced salt solution (EBSS) for 2 hours to enhance lysosomal function (41), followed by a 6-hour MazNP treatment. Flow cytometric analysis revealed that EBSS led to decreased surface azide expression in MazNP-treated cells compared with nontreated controls, while free Maz levels remained relatively unchanged (Supplemental Figure 19A). Interestingly, we observed that rhodamine fluorescence intensity from MazNP was markedly higher in tumor cells exposed to EBSS (Supplemental Figure 19B), suggesting that, while lysosomes were key for NP processing in both cell types, the specialized degradative function of macrophage lysosomes led to more effective degradation of MazNP than was seen in tumor cells.

### In vivo TRACER enhances tumor targeting and reduces liver accumulation.

**Figure 7:**
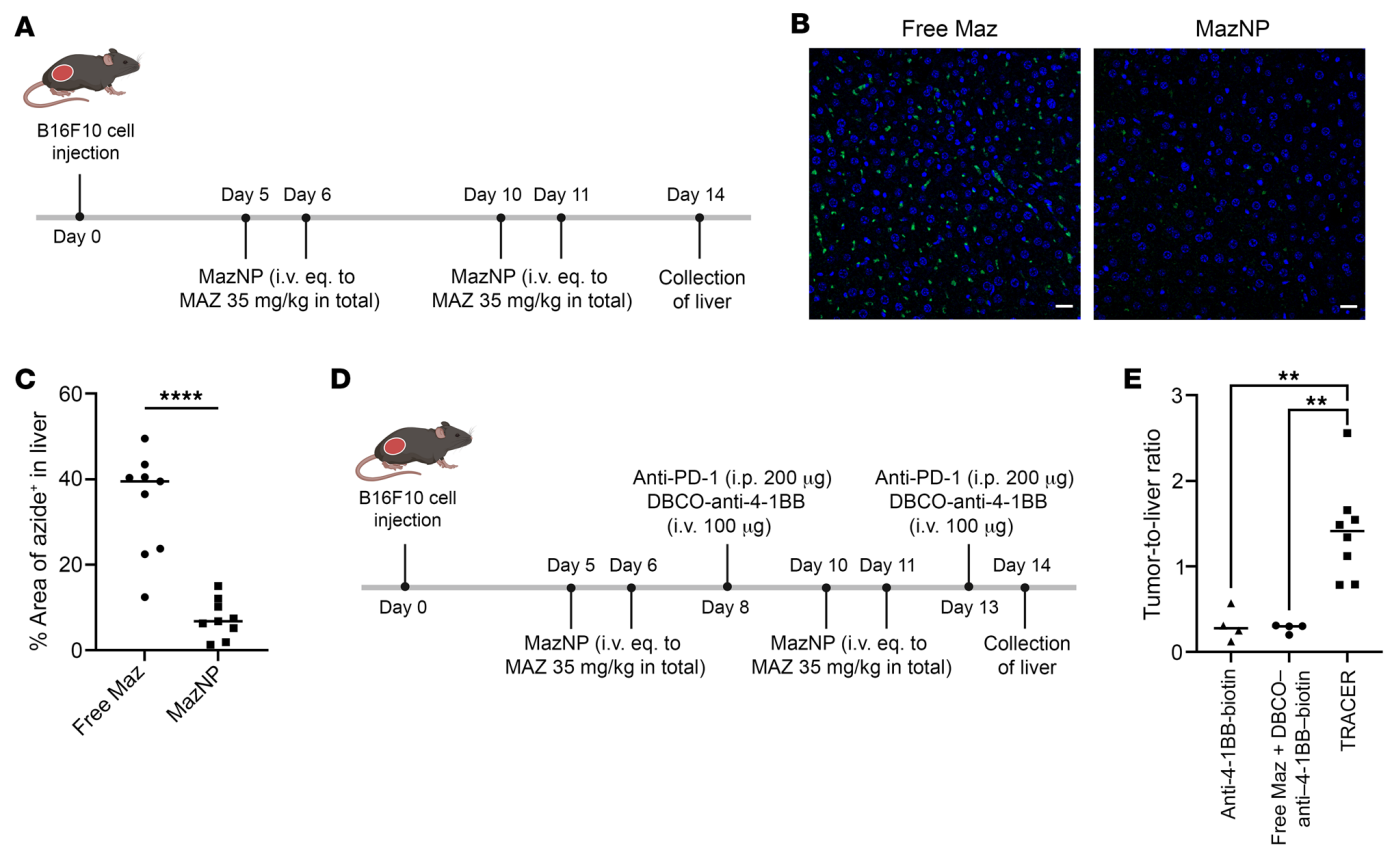
TRACER-mediated in vivo delivery of anti–4-1BB in C57BL/6 mice bearing B16F10 tumors. (**A**) Schematic of B16F10 tumor inoculation, treatments with free Maz or MazNP, and time points for liver collection. (**B**) Representative fluorescence images of liver tissue sections (green: DBCO-Cy5; blue: nuclei stained with Hoechst 33258) are shown again in Supplemental Figure 20. Scale bars: 20 μm. (**C**) Quantitative analysis of DBCO-Cy5–stained liver sections (*n* = 3 per group). The percentage of azide^+^ area was estimated as the area of the DBCO-Cy5^+^ area (green) divided by the area of the tissue in the field of view (outlined by blue Hoechst 33258 staining). Randomly selected fields (9 images per group; Supplemental Figure 20) were analyzed with Fiji software. ****P < 0.0001, by unpaired, 2-tailed *t* test. (**D**) Schematic of B16F10 tumor inoculation, treatments, and time points for tissue collection. Mice received anti–4-1BB–biotin (*n* = 4), free Maz plus DBCO–anti–4-1BB–biotin (*n* = 4), or DBCO–anti–4-1BB-biotin (*n* = 8) via the TRACER approach at specified time points. (**E**) Tumor/liver ratio of biotin-labeled Abs 24 hours after injection in B16F10 tumor–bearing mice. **P < 0.01, by Tukey’s multiple-comparison test following 1-way ANOVA.

To validate our in vitro findings, we assessed in vivo labeling efficiency of MazNP compared with free Maz. Mice received either free Maz or MazNP, similar to our toxicity studies (Figure 7A). We used DBCO-Cy5 staining to analyze liver (Figure 7B and Supplemental Figure 20) and tumor tissue sections (Supplemental Figure 21). Consistent with our in vitro findings, we observed significantly less azide labeling in the livers of mice that received MazNP (7.3% ± 4.5%) compared with those that received free Maz (34.3% ± 12.0%) (Figure 7C). MazNP-treated mice showed increased azide labeling (34.4% ± 21.6%) in tumors compared with those that received free Maz (1.4% ± 1.1%) (Supplemental Figure 21C). These in vivo findings validate the effectiveness of our TRACER targeting approach with MazNP in achieving precise in vivo labeling of tumor cells while minimizing off-target effects.

We further investigated whether TRACER increases the accumulation of anti–4-1BB Abs in tumors compared with the liver using biotin-labeled anti–4-1BB Abs (Figure 7D). B16F10 tumor–bearing mice received biotin-labeled anti–4-1BB or DBCO–anti–4-1BB via the TRACER approach. Twenty-four hours after treatment, the tumor, liver, kidneys, lungs, and spleen were harvested and homogenized. The tumor/liver ratio was significantly higher (5.1-fold and 4.5-fold, respectively) in the DBCO–anti–4-1BB plus MazNP group than in the DBCO–anti–4-1BB plus free Maz or anti–4-1BB groups (Figure 7E). In other organs, we observed that the MazNP-treated mice had significantly lower accumulation of DBCO–anti–4-1BB in the kidneys than did the free Maz-treated group and reduced accumulation in the lungs and spleen compared with both the free Maz and anti–4-1BB–only groups (Supplemental Figure 23B). These results demonstrate that the TRACER approach with MazNP effectively enhanced tumor-specific accumulation of anti–4-1BB, while reducing accumulation in nontumor tissues, potentially minimizing the systemic toxicities associated with anti–4-1BB therapy.

## Discussion

Targeted cancer therapy has traditionally relied on identifying and exploiting tumor-specific targets for antitumor treatment. While this targeting strategy represents a promising approach, its clinical translation has been hampered by a fundamental lack of tumor-specific targets or antigens across diverse tumor types, as well as by the heterogeneity of tumor expression. This heterogeneity necessitates tumor-specific evaluation of target expression and substantially limits the applicability of these therapies (12, 13). To address these limitations, we applied an NP-based TRACER delivery approach that utilizes metabolic glycoengineering combined with bioorthogonal click chemistry to uniformly express a tumor target therapeutically.

The preferential accumulation of MazNP in tumors is primarily attributed to the enhanced permeability and retention (EPR) effect (42, 43), which enables macromolecules, including NPs (<200 nm) (44–46), to accumulate more effectively in tumor tissue compared with conventional small molecules (44, 45, 47–49). By applying this strategy, we enabled tumor labeling with an Ab specific for 4-1BB, leading to antitumor T cell activation with very limited activation of T cells against nontumor tissue. This approach effectively overcame challenges related to limited tumor-specific targets.

Agonistic Abs targeting 4-1BB have shown potent immunomodulatory effects by enhancing T cell proliferation, survival, and effector function, thereby bolstering antitumor immunity (23, 50). However, the clinical application of 4-1BB agonists is limited because of severe hepatotoxicity. Studies have shown that 4-1BB agonists lead to liver inflammation and injury in patients, primarily due to the activation of type 1 CD8^+^ T cells within the liver (35, 51, 52). This off-target effect limits the therapeutic potential of agonistic anti–4-1BB Abs. Our TRACER platform addresses this crucial challenge by increasing the accumulation of anti–4-1BB Abs in tumors compared with the liver, which was associated with markedly decreased hepatotoxicity while preserving antitumor activity.

The tumor-specific targeting capability of our TRACER platform was further demonstrated by biodistribution studies, in which TRACER delivery achieved approximately 5.1- and 4.5-fold higher tumor/liver ratios of DBCO–anti–4-1BB when compared with non-TRACER delivery approaches. This enhanced tumor targeting was accompanied by reduced accumulation in other organs, such as the liver, kidneys, lungs, and spleen, highlighting TRACER’s ability to minimize off-target effects. These favorable distribution patterns offer mechanistic insight into both the enhanced therapeutic efficacy and reduced toxicity observed, particularly in traditionally resistant tumor models (53). The improved tissue selectivity achieved through TRACER delivery represents what we believe to be a significant advancement over conventional Ab delivery methods, supporting the broader potential of TRACER in cancer immunotherapy.

NP-based cancer therapeutics are known to be cleared by tissue macrophages and migrating monocytes (39, 40, 54–56). Thus, it was not initially clear that our approach, which uses MazNPs, would limit tissue toxicity. Surprisingly, our studies revealed that MazNP uptake by macrophages did not lead to cell-surface azide expression, unlike free Maz. This difference likely stems from distinct processing of MazNPs in lysosomes. Upon cellular entry via passive diffusion, free Maz was rapidly metabolized into *N*-azidoacetyl sialic acid (SiaNAz) for cell-surface expression (57), whereas NPs remained in lysosomes, where they underwent degradation (58). This lysosomal degradation in macrophages limited cell-surface expression, aligning with previous studies suggesting that drug-loaded NP uptake by liver macrophages is linked to reduced hepatotoxicity, in contrast to the effects observed with small-molecule drugs (59). We observed a similar trend when lysosomal activity was enhanced in B16F10 tumor cells, which had decreased surface azide expression from MazNP, suggesting that lysosomal degradation is a key mechanism in controlling cell-surface azide expression. These findings suggest that MazNP is processed in macrophages differently than Maz in tumor cells, leading to less anti–4-1BB accumulation in liver macrophages and reduced hepatotoxicity, while maintaining effective tumor targeting.

Recent studies have highlighted the accumulation of PMN-MDSCs in lymph nodes (LNs) during cancer progression. Their presence in LNs has been associated with poorer prognoses in gastric and bladder cancers (32, 33). These cells contribute to an immunosuppressive microenvironment by producing ROS, arginase, and cytokines, which can significantly suppress the cytotoxic activities of T cells and NK cells (60). In our study, we observed a notably decreased infiltration of PMN-MDSCs in TDLNs in the groups treated with TRACER-delivered anti–4-1BB with MazNP compared with those that received nontargeted anti–4-1BB. By preferentially directing anti–4-1BB to the tumor site, TRACER platform has the potential to enhance therapeutic outcomes.

Our TRACER approach offers several potential advantages over existing 4-1BB–targeting strategies. Previous studies involving 4-1BB agonistic Abs such as urelumab demonstrated significant hepatotoxicity (26), while utomilumab showed limited efficacy (61). In contrast, TRACER can overcome these issues by localizing anti–4-1BB to tumor sites, potentially enhancing both safety and efficacy. Unlike bispecific Abs, such as DuoBody-PD-L1×4-1BB (GDN1046) (62) and the human×PD-L1 bispecific Ab (MCLA-145), which rely on PD-L1 expression for tumor targeting (63), TRACER’s NP-based delivery system allows for tumor targeting independent of the expression of specific tumor proteins, potentially broadening its applicability across various tumor types. Additionally, TRACER is particularly advantageous for agents that do not require cellular uptake. It is worth noting that, with its 2-step process, TRACER involves initial delivery of MazNP followed by administration of DBCO-functionalized Abs and therefore presents challenges for clinical translation, including optimizing the timing between steps and ensuring consistent biodistribution of both components. Despite these limitations, TRACER’s demonstrated ability to enhance tumor-specific targeting while reducing systemic toxicity, along with its flexibility in delivering various immunotherapeutic agents, makes it a promising approach for advancing cancer immunotherapy. For example, TRACER could potentially be used to deliver anti–CTLA-4 to block immune checkpoints, anti-OX40 to provide costimulatory signals, or agents to impede Treg-mediated immune suppression in the TME.

In conclusion, our study highlights the substantial potential of the TRACER approach to advance cancer immunotherapy. By circumventing the need to identify tumor-specific markers, TRACER enables selective tumor targeting while minimizing off-target effects and hepatotoxicity. The unique processing of MazNP in macrophage lysosomes contributes to the redistribution of Maz accumulation, enhancing tumor-specific delivery of anti–4-1BB. This strategy shows promise for treating various cancers, particularly those lacking specific surface markers such as pancreatic and triple-negative breast cancers, and it may potentially re-enable clinical studies using anti–4-1BB to treat tumors. Continued research and optimization of the TRACER platform could lead to a paradigm shift in cancer therapy, offering more targeted, safer, and effective treatments that improve patient outcomes.

## Methods

### Sex as a biological variable.

This study exclusively utilized female mice for both the B16F10 melanoma (64, 65) and 4T1 breast cancer (66, 67) models, consistent with established research protocols and prior studies. We focused on female mice, aged 7–8 weeks, to ensure experimental consistency and reproducibility, given the significant sex-specific disparity in the incidence of breast cancer. Sex was not considered a biological variable in the design of this study. While our findings are pertinent to females, further research is required to determine their applicability to males and to assess whether the observed effects are sex specific.

Additional details on methods and materials can be found in the Supplemental Methods.

### Study design.

The objective of this study was to develop an NP-based, tumor-specific surface marker–independent targeting approach to overcome challenges related to unreliable tumor markers. As shown in the figure legends, all experiments were repeated, and no experimental data were excluded from the quantitative analysis. In our in vivo studies, tumor-bearing mice were randomized into groups according to tumor size 1 day prior to treatment. Tumor volume was assessed by 2 independent researchers, with 1 researcher blinded to treatment group assignments. Mice were monitored daily and euthanized at predefined humane endpoints. All animal studies involved 7- to 8-week-old female C57BL/6 mice or BALB/c mice (The Jackson Laboratory). The antitumor efficacy of Abs with or without MazNP was evaluated in B16F10 melanoma (*n* = 8 or 11 per group) or 4T1 orthotopic tumor models (*n* = 8 per group). Statistical differences in average tumor growth curves were analyzed using 2-way ANOVA with time and tumor volume as variables. Survival differences across groups were assessed using the Kaplan-Meier method, with the overall *P* value calculated by the log-rank test using GraphPad Prism 6.0. Immune cell populations in B16F10 tumors (*n* = 3 or 4 per group) were examined using flow cytometry and IF staining, and confocal microscopy (9 images per group) was analyzed with Fiji software. TDLN samples were analyzed for immune cell populations using flow cytometry. Depletion studies were conducted using B16-F10 tumor–bearing mice (*n* = 8 per group). Additionally, in vivo labeling of Maz in liver and tumor tissues was assessed using DBCO-Cy5–stained tissue sections from B16F10 tumor–bearing mice (*n* = 3 per group). Analysis was conducted on randomly selected fields from 9 images per group. Liver and tumor labeling of Maz in B16F10 mice (*n* = 3 per group) was assessed using DBCO-Cy5–stained tissue sections. Hepatotoxicity studies involved IF, IHC (*n* = 3 per group; 3 to 8 images per tissue), and flow cytometry (*n* = 3 or 4 per group) for liver immune cells. Serum liver enzyme and cytokine levels were analyzed (*n* = 8 per group).

### Statistics.

All statistical analyses were performed using GraphPad Prism 9 (GraphPad Software). All data are presented as the mean ± SD. Statistical significance was determined by 1-way ANOVA with Tukey’s or Dunnett’s multiple-comparison test, 2-way ANOVA with Šidák’s multiple-comparison test, or unpaired, 2-tailed *t* test. Survival curves were analyzed using the Kaplan-Meier method and the log-rank (Mantel-Cox) test. All image analyses were performed using Fiji software (NIH). A *P* value of less than 0.05 was considered significant.

### Study approval.

All animal work was conducted in accordance with protocols (19-001.0 and 23.055.0), which were approved and monitored by the IACUC of UNC.

### Data availability.

The underlying data in this work are available from the corresponding author upon request. Values for all data points in graphs are reported in the Supporting Data Values file.

## Author contributions

HH, BS, JSS, and AZW conceived and designed the research study. HH, BS, MY, AW, and YW conducted the experiments. HH, BS, MY, and AW acquired the data. HH, BS, MY, SAM, TZ, JC, JSS, and AZW analyzed and interpreted the data. HH drafted the initial manuscript, and all authors reviewed and contributed to its editing. AZW supervised the research.

## Supplementary Material

Supplemental data

Supporting data values

## Version 1 03/11/2025

In-Press Preview

## Version 2 05/01/2025

Electronic publication
