# Supplementary material for: Tumor-specific surface marker–independent targeting of tumors through nanotechnology and bioorthogonal glycochemistry
Source: J Clin Invest. 2025 Mar 11;135(9):e184964. doi: 10.1172/JCI184964 (PMC12043094; doi:10.1172/JCI184964)
Supplement: Supplemental data [file jci-135-184964-s092.pdf]

Supplemental Materials and Methods

Reagents

mPEG-PLGA (AK102; LA:GA = 50:50 (w:w);M_W: ≈5000:30,000 Da) was obtained from Akina Inc. (West Lafayette, IN). N-azidoacetylmannosamine-tretraacylated (Ac_4ManNAz), DBCO-PEG4-biotin, DBCO-Cy5 were purchased from Click Chemistry Tools (Scottsdale, AZ). DBCO-PEG_{13}-NHS ester (M_W = 1046.2 g/mol) was purchased from BroadPharm (San Diego, CA). Anti-PD-1 (clone: RMP1-14), anti-4-1BB (CD137) (clone: 3H3), anti-CD8a (clone: 2.43), anti-NK1.1 (clone: PK136), and Fc Block (clone: 2.4G2) were purchased from BioXCell (West Lebanon, NH). SpectraPro® Float-A-Lyzer® G2 dialysis device was obtained from REPLIGEN (Waltham, MA). FITC-Streptavidin was purchased from BioLegend (San Diego, CA). Recombinant Mouse 4-1BB/TNFRSF9 Fc Chimera Protein and Enzyme-linked immunosorbent assay (ELISA) kits for mouse TNF-α, IL-6, and IFN-γ were purchased from R&D Systems (Minneapolis, MN). Goat anti-rat IgG (H+L) secondary antibody horseradish peroxidase (HRP), 1-step ultra TMB-ELISA substrate solution, stop solution for TMB Substrates, MaxiSorp flat-bottom plates (NUNC brand products), Zeba Spin 7K MWCO Desalting Columns, and BCA protein assay were from Fisher Scientific. All other chemicals were obtained from Sigma-Aldrich unless otherwise noted.

Cell lines

B16F10 and 4T1 cell lines were acquired from ATCC, where they were authenticated using morphology, karyotyping, and polymerase chain reaction (PCR)-based approaches and tested for mycoplasma. B16F10 cells were cultured in Dulbecco's modified Eagle's medium (DMEM) (Gibco) supplemented with 10% fetal bovine serum (Mediatech) and antibiotic-antimycotic (Anti-Anti: 100 U of penicillin, 100 μg/ml of streptomycin and 0.25 μg/ml of amphotericin B; Gibco). 4T1 cells were cultured in RPMI Medium 1640 (Gibco) supplemented with 10% fetal bovine serum (Mediatech) and Anti-Anti (1×). J774A.1 mouse macrophages (ATCC) were grown in Dulbecco's modified Eagle medium (DMEM) supplemented with 10% FBS, 100 units/mL of penicillin and 100 mg/mL of streptomycin. Cell cultures were maintained below 50% confluence and early-passage cultures (between 5 and 8) were utilized for experiments.

Preparation of Maz-loaded NP

NPs were prepared by a nanoprecipitation method, chosen for its reproducibility and ability to generate uniform particles. First, mPEG-PLGA (LA:GA = 50:50 (w:w);Mw: ≈5000:30,000 Da) was dissolved into acetonitrile (ACN) with a final polymer concentration of 10 mg/ml. The organic phase was added dropwise into the aqueous phase (endotoxin free H2O) through a syringe under an oil to

water ratio of 1:3. The solution was stirred at room temperature under a vacuum until the ACN completely evaporated. The NPs were collected and washed three times with endotoxin-free H2O using ultrafiltration (Amicon Ultra Centrifugal Filter Units, 100,000 MWCO). When Maz was loaded, Maz was incorporated into the organic phase along with PLGA (1:4 weight ratio), with a final polymer concentration of 10 mg/ml at the theoretical loading 20 wt%. The Maz-loaded NPs (MazNP) were washed in the same manner as blank NPs.

Characterization of MazNP

NPs were prepared as a 1 mg/ml suspension in 10 mM NaCl. The size, PDI (1), and zeta potential (2) of the produced NPs were measured using a Malvern Zetasizer Nano ZS instrument (Malvern, Inc.). NPs were stained with 2 % uranyl acetate solution and imaged by TEM using Thermofisher Talos F200X at an accelerating voltage of 200kV. The Maz loading efficiency, defined as loaded MazNP mass, was determined by High Performance Liquid Chromatography (HPLC) (Supplemental Figure 2). NPs with a known mass were dissolved in 0.5 ml of ACN. After 30 min, polymers were precipitated with the addition of 0.5 ml of deionized (DI) water, centrifuged, and the supernatant was filtered with a 0.45 mm syringe filter. The samples were analyzed via a Shimadzu SPD-M20A HPLC and equipped with a C8 column. A linear gradient of 20% to 50% mobile phase B (ACN with 0.1% TFA) for 30 min was performed at a flow rate of 0.5 ml/min. Sample injection volume was 50 µL and UV absorbance was monitored at 280 nm.

Preparation of DBCO-functionalized anti-4-1BB

DBCO-functionalized anti-4-1BB (DBCO-anti-4-1BB) was synthesized via a primary amine N-hydroxysuccinimide (NHS) coupling reaction between NHS ester-activated DBCO ligand and primary amines in the antibody. One milligram of anti-4-1BB in PBS was mixed with 0.14, 0.24, and 0.33 µmol of DBCO-PEG13-NHS (47.79 mM in DMSO) for the target DBCO to anti-4-1BB molar ratios were 20:1, 35:1, and 50:1, respectively. The mixture was diluted to a final antibody concentration of 5 mg/ml and incubated under horizontal shaking at 100 rpm for 2 h at room temperature in the dark. The DBCO-anti-4-1BB solution was then purified via dialysis using a Float-a Lyzer dialysis device (8-10 kD cutoff) against PBS at 4 °C for three days. The concentration of the purified DBCO-anti-4-1BB was determined by a BCA protein assay and stored at 4 °C for further studies.

Characterization of DBCO-functionalized anti-4-1BB

The DOF (the number of DBCO conjugated to anti-4-1BB) of different DBCO-functionalized anti-4-1BB were determined via a UV-vis absorption spectroscopy method (3). Briefly, the concentrations and degrees of the DBCO incorporation of different DBCO-conjugated anti-4-1BB were determined

spectroscopically and calculated using the molar extinction coefficient of DBCO at 310 nm = 12,000 M^{-1} cm^{-1}, correction factor of DBCO at 280 = 1.089, extinction coefficient of anti-4-1BB at 280 nm = 1.33, and the molar extinction coefficient of anti-4-1BB at 280 nm = 210,000 M^{-1} cm^{-1} according to the manufacturer's instructions. The DOF of different DBCO-functionalized anti-4-1BB were further confirmed with AB Sciex 5800 matrix-assisted laser desorption ionization-time-of-flight mass spectrometer (MALDI-TOF MS) at the UNC Michael Hooker Proteomics Center as previously published (4). Briefly, antibody samples were dialyzed against deionized water at 4 °C for 36 h (6 cycles) using 8-10 kDa cutoff Float-A-Lyzer G2 dialysis devices. Desalted samples (1 mg/ml) were loaded on a Teflon-coated plate with sinapinic acid as the matrix after a five-fold dilution with 50% methanol containing 0.1% formic acid (FA). Samples were laser-irradiated with 500 shots, and the molecular weight was determined by following the standard MALDI-TOF MS method using peptide calibration mixture 4700 (AB Sciex) and BSA. Using the MALDI molecular weights (MWs) of DBCO-anti-4-1BB, the number of DBCO conjugated to anti-4-1BB was estimated through the following equation (5).

$$\text{DBCO linker : anti-4-1BB} = \frac{\mathrm{MW_{mconj}} - \mathrm{MW_{\alpha 4-1BB}}}{(\mathrm{MW_{DBCO\ linker}} - 115)}$$

Where MW_{mconj}, $MW_{anti\text{-}4\text{-}1BB}$, and $MW_{DBCO\text{-}linker}$ are the MWs of the final DBCO-anti-4-1BB, anti-4-1BB, and DBCO-PEG_{13}-NHS ester, respectively, and 115 is the MW of the departing NHS after DBCO-antibody coupling.

To examine if DBCO conjugation affects the binding properties of anti-4-1BB, ELISA was performed (6). Recombinant mouse 4-1BB/TNFRSF9 Fc chimera proteins were immobilized (2 ug/ml) on Maxisorp plates (NUNC Brand Products) overnight at 4 °C. After washing and blocking, DBCO-functionalized antibodies with different concentrations were added and incubated for 1 h at room temperature. After washing, 200 ng/ml of HRP-conjugated goat anti-rat IgG was then added as the detection antibody, followed by an HRP-sensitive colorimetric substrate. The absorbance of ELISA test results was read at 450 nm.

Efficacy of TRACER in improving tumor immunotherapy

In the melanoma tumor model, 75,000 B16F10 cells were suspended in DMEM, mixed with an equal volume of Matrigel (BD Biosciences), and subcutaneously inoculated on the right flank of C57BL/6 mice on day 0. B16F10 tumor-bearing mice were randomized into 6 groups (n = 8 per group) and treated with intravenous (IV) injections of PBS, free Maz (17.5 mg/kg), or MazNP (eq. to Maz 17.5 mg/kg) on day 5, 6, 10, and 11. 200 µg anti-PD-1 was intraperitoneally (IP) and 100 µg anti-4-1BB or DBCO-anti-4-1BB was intravenously (IV) injected into animals on day 8 and 13. To examine the

impact of the administration of Maz by NP, a control group where mice were given free Maz prior to DBCO-anti-4-1BB was included. In the breast tumor model, 100,000 4T1 cells were suspended in RPMI Medium 1640, mixed with an equal volume of Matrigel, and injected on the left fourth mammary fat pad of BALB/c mice (8-week-old female) on day 0. 4T1 tumor-bearing mice were treated with antibodies in the same manner as B16F10 tumor-bearing mice. Two days later, when the tumors reached an average size of ~80 mm^3, the animals were given anti-4-1BB or DBCO-anti-4-1BB. Tumor growth was monitored daily until the end point was reached. The length (L) and width (W) of each tumor was measured by a digital caliper, and the volume (V) was calculated by the modified ellipsoid formula: $V = (L \times W^2)/2$ (7). In the depletion study, mice were treated with anti-PD-1 plus DBCO-anti-4-1BB with MazNP using the same procedure. 400 μg per dose of anti-CD8a or anti-NK1.1 was injected intraperitoneally (IP) on day 14 (one day after the last treatment) (8).

Flow cytometric analysis

The immune profiles of tumor, liver, and TDLN from B16F10-tumor bearing C57BL/6 mice 18 days post-inoculation. Mice were inoculated subcutaneously with 75,000 B16F10 cells suspended in DMEM, mixed with an equal volume of Matrigel on the right flank. Post-inoculation, mice were randomized into four groups and received IV injections of PBS, free Maz (17.5 mg/kg) or MazNP (eq. to Maz 17.5 mg/kg) on day 5, 6, 10, and 11. Two hundred micrograms anti-PD-1 was intraperitoneally (IP) and 100 μg anti-4-1BB or DBCO-anti-4-1BB was intravenously (IV) injected into animals on day 8 and 13. Organs were collected five days after the final dose injection and processed to obtain single-cell suspensions through mechanical and enzymatic digestion. Initially, resected tumors and livers were sectioned into small pieces using a razor. Subsequently, tissues underwent digestion using collagenase, DNase I, hyaluronidase (Sigma-Aldrich), and Liberase (Roche) for 45 min in a gently stirred incubator at 37°C. The digested tissues were then filtered through a 70 μm cell strainer, washed with staining buffer, and resuspended in 1 ml ammonium-chloride-potassium buffer to lyse red blood cells. The cells were washed with PBS and stained with fixable live/dead stain for 30 min on ice. Following washing, cells were incubated for an additional 20 min on ice in Fc Block (clone 2.4G2; BioXCell) solution, and subsequently stained with a cocktail of fluorescently labeled antibodies for 25 min on ice. The cells were fixed with a 2% PFA solution and analyzed using the BD FACSymphony™ A3 Cell Analyzer equipped and FACSDiva software. Collected data were analyzed by FlowJo software (TreeStar, Ashland, OR). Detailed information on the antibodies and gating strategies used is provided in Supplemental Table 5 and Supplemental Figure 4.

Immunofluorescence staining

$CD3^+$, $CD4^+$, and $CD8^+$ T cells were analyzed in tumors from B16F10 tumor-bearing C57BL/6 mice 18 days post-inoculation, following the same timeline as the flow cytometry analysis study. Five days after the final dose injection, mice were sacrificed, and tissues were fixed in 10% neutral buffered formalin for 48 h, then transferred to 70% ethanol. Tissue sections (5 µm) were processed using the Bond fully-automated slide staining system (Leica Microsystems Inc., Norwell, MA) with the Bond Research Detection System kit (DS9455, Leica). Deparaffinization and rehydration were performed using Leica Bond Dewax solution (AR9222, Leica Biosystems Newcastle Ltd, United Kingdom) and Bond Wash solution (AR9590, Leica Biosystems Newcastle Ltd). Triple staining for CD3 (1:400; 85061S, Cell Signaling, Danvers, MA), CD4 (1:100; 14-9766-82, Invitrogen, Eugene, OR), and CD8a (1:400; 14-0808-80, Invitrogen) was conducted, including heat-induced epitope retrieval in Bond-epitope retrieval solution 1 pH 6.0 (AR9961, Leica Biosystems Newcastle Ltd.) at 100 °C. After blocking in in Background Sniper, the sections were treated with ImmPRESS goat anti-rat IgG treatment (Vector Laboratories, Newark, CA, USA). Staining utilized Cyanine 5 (FP1117, Akoya Biosciences), Cyanine 3 (FP1046, Akoya Biosciences), and Alexa Fluor 488 (B40953, Invitrogen) Tyramide reagents. Dual staining of liver sections was performed using antibodies against CD163 (1:500; ab182422, abcam) and CD206 (1:1000; ab64693, abcam), followed by incubation in Novolink Polymer and staining with Cyanine 3 and Cyanine 5 Tyramide Reagents, using the same process as the triple IF staining. Nuclei were counterstained with Hoechst 33258 (H3569, Invitrogen), and slides were mounted with ProLong Gold antifade reagent (P36930, Life Technologies). Imaging was performed on a Zeiss LSM 700 confocal microscope at the UNC School of Medicine Microscopy Services Laboratory Core Facility. Quantitative analysis was carried out using Fiji software (National Institute of Health, Bethesda, MD) in 3 random fields per tissue section.

Immunohistochemical staining

B16F10 tumor-bearing mice were treated the same as in IF staining. Chromogenic immunohistochemistry (IHC) was performed on paraffin-embedded tissues that were sectioned at 5 µm. IHC was carried out using the Leica Bond III Autostainer system. Slides were deparaffinized in Bond Dewax solution and hydrated in Bond Wash solution. Heat-induced biomarker retrieval was performed in Bond-epitope retrieval solution 1 pH 6.0 (Leica Biosystems Newcastle Ltd.), and nonspecific binding was blocked by incubation in Background Sniper. After pretreatment, slides were incubated with mouse anti-CD8 (14-0808-80, ebioscience, San Diego, CA) solution at 1:1,000 for 1h followed by ImmPRESS goat anti-rat IgG (Vector Laboratories) for 30 min as the secondary. Antibody detection with 3,3'-diaminobenzidine (DAB) was performed using the Bond Intense R detection system (Leica Biosystems Newcastle Ltd.). Stained slides were dehydrated and coverslipped with Cytoseal 60 (8310-4, Thermo Fisher Scientific). Images were taken with an

Olympus BX61 microscope. The CD8-stained area in the liver was quantified by Fiji image analysis software (National Institute of Health, Bethesda, MD) in 3 to 8 random fields per tissue section.

Toxicity studies

B16F10 tumor-bearing mice were treated the same as in IF staining. Five days after the last dose of antibodies, mice were euthanized and livers and spleens were surgically removed, weighed, and fixed in 10% neutral buffered formalin for 48 h and then transferred to 70% ethanol. The fixed tissues were then embedded in paraffin, sectioned (4 μm), and stained with hematoxylin and eosin for histological evaluation. Blood was collected via cardiac puncture, allowed to clot in a silica-coated tube, and submitted to Pathology Services Core at the University of North Carolina-Chapel Hill for analysis of alanine aminotransferase (ALT) and aspartate aminotransferase (AST) serum levels. Serum levels of cytokines (TNF-α, IL-6, and IFN-γ) were measured by ELISA in accordance with the manufacturer's protocol.

In vitro labeling of Maz

Fluorescently labeled/non-PEGylated MazNPs were prepared with PLGA-rhodamine B (50:50, 10 – 30k; AV011, Akina, IN). J774A.1 mouse macrophages or B16F10 cells were plated in a 35 mm glass-bottomed dish at a density of 30,000 cells per well. After 24 h, the old medium was discarded, and cells were incubated with free Maz (50 μM) or MazNP (eq. 50 uM Maz) in the serum-supplemented medium at 37 ˚C for 6 h. After 6 h, cells were gently washed or incubated for an additional 18 h in the fresh serum-supplemented medium (24 h). After washing with PBS, cells were incubated with DBCO-PEG4-biotin (100 μM) in the serum-free medium for 1 h at 37 ˚C. The cells were gently rinsed again and incubated with 5 μl of FITC-streptavidin (1:200 dilution) in the serum-free medium for 40 min at 37 ˚C. For MazNP tracking, cells were rinsed and treated with 50 nM LysoTracker Deep Red (Invitrogen) at 37 ˚C for 25 min. Nuclei were counterstained with NucBlue Live ReadyProbes (Hoechst 33342, Invitrogen) according to the manufacturer's protocol. Cells were washed with PBS, fixed with 4% paraformaldehyde (PFA) (Alfa Aesar, Tewksbury, MA) in PBS for 10 min at room temperature, and then washed with DPBS (pH 7.4). To inhibit lysosomal activity, J774A.1 macrophages were pretreated with chloroquine (50 μM) for 1 h, washed twice, and incubated with rhodamine-labeled/non-PEGylated MazNPs for 6 h. After washing with PBS, cells were treated with DBCO-PEG4-biotin (100 μM) for 1 h, followed by 5 μl of FITC-streptavidin for 40 min at 37 ˚C. Cells were imaged with a Zeiss LSM 900 spectral confocal laser scanning microscope in the Microscopy Services Laboratory Core Facility at the UNC School of Medicine.

For flow cytometry analysis, J774a.1 macrophages were treated as described for confocal microscopy. After 1, 6, and 24 h post-treatment, cells were gently washed and incubated with DBCO-PEG4-biotin

(100 µM) in the serum-free medium for 1 h at 37 ˚C. The cells were harvested by scraping, collected by centrifugation at 233 rcf for 3 min, incubated with 5 µ of FITC-streptavidin (1:200 dilution) in PBS for 40 min at 37 ˚C. The cells were washed with PBS and stained with live/dead fixable violet stain (Invitrogen) for 30 min on ice. Sample acquisition was performed using a MACS Quant (Miltenyi Biotec), and data were analyzed by FlowJo v10 (TreeStar, BD) and Prism v10 (GraphPad). B16F10 cells were pre-incubated with Earle's Balanced Salt Solution (EBSS) for 2 h to enhance lysosomal activity, followed by incubation with free Maz or MazNP for an additional 6 h, treated in the same manner as above, and then analyzed using a MACS Quant.

In vivo liver and tumor labeling of Maz

B16F10 tumor-bearing mice were treated with IV injections of free Maz (17.5 mg/kg) or MazNP (eq. to Maz 17.5 mg/kg) on day 5, 6, 10, and 11. 24 h after the final dose of free Maz or MazNP, mice were euthanized, and livers and tumors were surgically removed and fixed in 10% neutral buffered formalin for 48 h and then transferred to 70% ethanol. The fixed tissues were then embedded in paraffin, sectioned (5 µm), and stained with hematoxylin and eosin for histological evaluation. Staining was carried in the Bond fully automated slide staining system (Leica Microsystems). Slides are dewaxed in Bond Dewax solution and hydrated in Bond Wash solution. Pretreatment is 5 min Bond peroxide (DS9800) and 30 min protein block (BS966MM, Biocare Medical, Pacheco, PA). After pretreatment, slides are incubated with DBCO-Cy5 for 1 h. DBCO-Cy5 was constituted by 200 µl DMSO and 1:1000 diluted by DI water. Nuclei were counterstained with Hoechst 33258 and mounted with ProLong Gold antifade reagent (P36934, Life Technologies). Images were taken with a Zeiss LSM 900 spectral confocal laser scanning microscope in the Microscopy Services Laboratory Core Facility at the UNC School of Medicine.

Tissue distribution of biotin-labeled DBCO-functionalized anti-4-1BB

The concentrations of anti-4-1BB antibody and DBCO-anti-4-1BB antibodies in tissues, including tumor, liver, kidney, lung, and spleen, were quantified using ELISA. The antibodies were biotinylated through amine-NHS ester reaction with biotin-PEG4-NHS Ester (CCT-B103, Vector Laboratories), according to the manufacturers' instructions. UV-visible absorbance spectroscopy confirmed successful conjugation, showing distinct peaks at 310 nm and 354 nm corresponding to DBCO and biotin, respectively (Supplemental Figure 22A). The molar substitution ratio (MSR) for biotin was established at 1.3, as determined by UV-visible spectroscopy using the E1% ChromalLINK Biotin MSR Calculator (Vector Laboratories). To ensure functionality, ELISA was used to validate the biotin-labeled antibodies prior to their use in quantifying tissue concentrations, demonstrating a linear response with increasing concentrations of biotin-labeled antibodies

(Supplemental Figure 22B). Tissues were homogenized in PBS containing cOmplete™ EDTA-free Protease Inhibitor Cocktail (11836170001, Roche, Basel, Switzerland) using a FastPrep-24 Classic bead beating grinder and lysis system (MP Biomedicals, Irvine, CA). The homogenates were centrifuged at 21,130 x g for 15 min at 4 ˚C, and the supernatants were collected. Protein concentrations in the supernatants were determined using the Pierce BCA Protein assay kit. For ELISA, recombinant mouse 4-1BB/TNFRSF9 Fc chimera protein (937-4B-050, R&D Systems, Minneapolis, MN) was coated onto Maxisorp ELISA plates (NUNC Brand Products) at (3 µg/ml) and incubated overnight at 4 ˚C (39). Plates were then washed and blocked, followed by the addition of the tissue homogenates for a 1-hour incubation at room temperature. To accurately quantify the antibody concentrations in specific tissues, standard curves were prepared for each tissue type. anti-4-1BB-biotin or DBCO-anti-4-1BB-biotin was mixed with homogenates from corresponding non-treated B16F10 tumor-bearing mouse tissues. The wells were washed and HRP-streptavidin (1:2,000; SA-5014-1, Vector Laboratories) was added. The color development on the plates was carried out using TMB solution (eBioscience), reaction was terminated with stop solution (Invitrogen). Absorbance was measured at 450-570 nm.

Supplemental Table 1. Size, zeta potential and Maz-loading efficiency of MazNP

**NP type**	**z-average (d.nm)**	**Polydispersity index (PDI)** [a]	**Zeta potential (mV)** [b]	**Maz loading efficiency (LE %)**
NP	98 ± 8	0.11 ± 0.02	-3 ± 1	N/A
MazNP	119 ± 4	0.21 ± 0.07	-8 ± 2	6.3 ± 0.8

[a] Polydispersity index (PI), an estimate of particle size distribution width, obtained from the cumulant analysis as described in the International Standard on DLS ISO 13321:1996 and ISO 22412:2008 (Malvern DLS technical note MRK176401). PI < 0.1 is considered monodisperse, and >0.7 is very broad (1, 2).

[b] Zeta potential measured in 10 mM NaCl.

n = 6 identically and independently prepared samples (mean ± s.d.).

Supplemental Table 2. Size and PDI of MazNP at different concentrations (1, 10, 25, and 50 mg/mL)

**Concentration (mg/mL)**	**1**	**10**	**25**	**50**
**z-average (d.nm)**	118.0 ± 3.3	122.9 ± 7.2	132.6 ± 1.0	134.0 ± 3.3
**Polydispersity index (PDI)**	0.17 ± 0.01	0.20 ± 0.02	0.16 ± 0.01	0.17 ± 0.01

n = 3 identically and independently prepared samples (mean ± s.d.).

Supplemental Table 3. Size of MazNP in 50% FBS

**z-average (d.nm) in 50% FBS**	129.6 ± 1.0
**Polydispersity index (PDI)**	0.23 ± 0.03

NPs were suspended in 50% FBS to 10 mg/mL. The size and PDI of the MazNP were measured by a Malvern Zetasizer Nano ZS instrument.

n = 3 identically and independently prepared samples (mean ± s.d.).

Supplemental Table 4. Statistics comparing the Kaplan-Meier survival curves of B16F10 bearing mice

**Comparison group**	***P*-value (log-rank test)**
PBS vs anti-PD-1	0.0014
PBS vs anti-PD-1+anti-4-1BB	0.0001
PBS vs anti-PD-1+DBCO-anti-4-1BB	<0.0001
PBS vs anti-PD-1+DBCO-anti-4-1BB+free Maz	<0.0001
PBS vs anti-PD-1+TRACER	<0.0001
anti-PD-1 vs anti-PD-1+anti-4-1BB	0.1997
anti-PD-1 vs anti-PD-1+DBCO-anti-4-1BB	0.3197
anti-PD-1 vs anti-PD-1+DBCO-anti-4-1BB+free Maz	0.0308
anti-PD-1 vs anti-PD-1+TRACER	0.0019
anti-PD-1+anti-4-1BB vs anti-PD-1+DBCO-anti-4-1BB	0.3485
anti-PD-1+anti-4-1BB vs anti-PD-1+DBCO-anti-4-1BB+free Maz	0.7315
anti-PD-1+anti-4-1BB vs anti-PD-1+TRACER	0.0071
anti-PD-1+DBCO-anti-4-1BB vs anti-PD-1+DBCO-anti-4-1BB+free Maz	0.1214
anti-PD-1+DBCO-anti-4-1BB vs anti-PD-1+TRACER	0.0071
anti-PD-1+DBCO-anti-4-1BB+free Maz vs anti-PD-1+TRACER	0.0697

P-values between groups were calculated by the log-rank test.

Supplemental Table 5. List of antibodies used in flow cytometry analysis

Antibody	Manufacturer	Clone	Catalog number
Zombie NIR™ Fixable Viability Dye	BioLegend		423105
FITC anti-mouse CD45	BioLegend	30-F11	103108
PE/Cyanine5 anti-mouse CD19	BioLegend	6D5	115509
APC anti-mouse CD49b	BioLegend	DX5	108910
PE/Cyanine7 anti-mouse NK-1.1	BioLegend	PK136	108714
BUV496 Rat Anti-Mouse CD4	BD Biosciences	GK1.5	612952
BUV395 Rat Anti-Mouse CD8a	BD Biosciences	53-6.7	563786
Brilliant Violet 711 anti-mouse/human CD44	BioLegend	IM7	103057
CD62L (L-Selectin) Monoclonal Antibody (MEL-14), PE-eFluor 610	eBioscience	MEL-14	61-0621-82
Brilliant Viole 785™ anti-mouse Ly-6C	BioLegend	HK1.4	128041
BUV805 Rat Anti-Mouse Ly-6G	BD Biosciences	1A8	741994
Alexa Fluor® 700 anti-mouse/human CD11b	BioLegend	M1/70	101222
PE anti-mouse CD11c	BioLegend	N418	117308
Brilliant Violet 421 anti-mouse F4/80	BioLegend	BM8	123137
Brilliant Violet 650 anti-mouse I-A/I-E	BioLegend	M5/114.15.2	107641

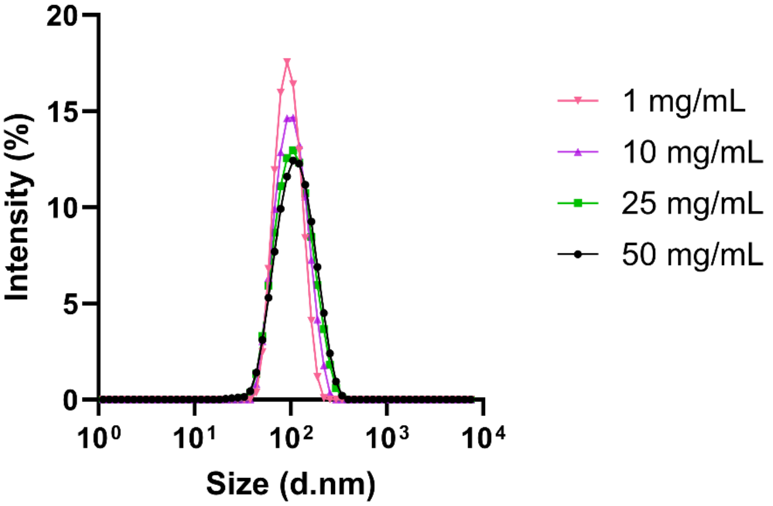

Supplemental Figure 1. Size distribution of MazNP at various concentrations. Intensity-based size distribution of MazNP measured by DLS at concentrations of 1, 10, 25, and 50 mg/mL (*n* = 3). Samples were prepared identically and independently (mean ± s.d.).

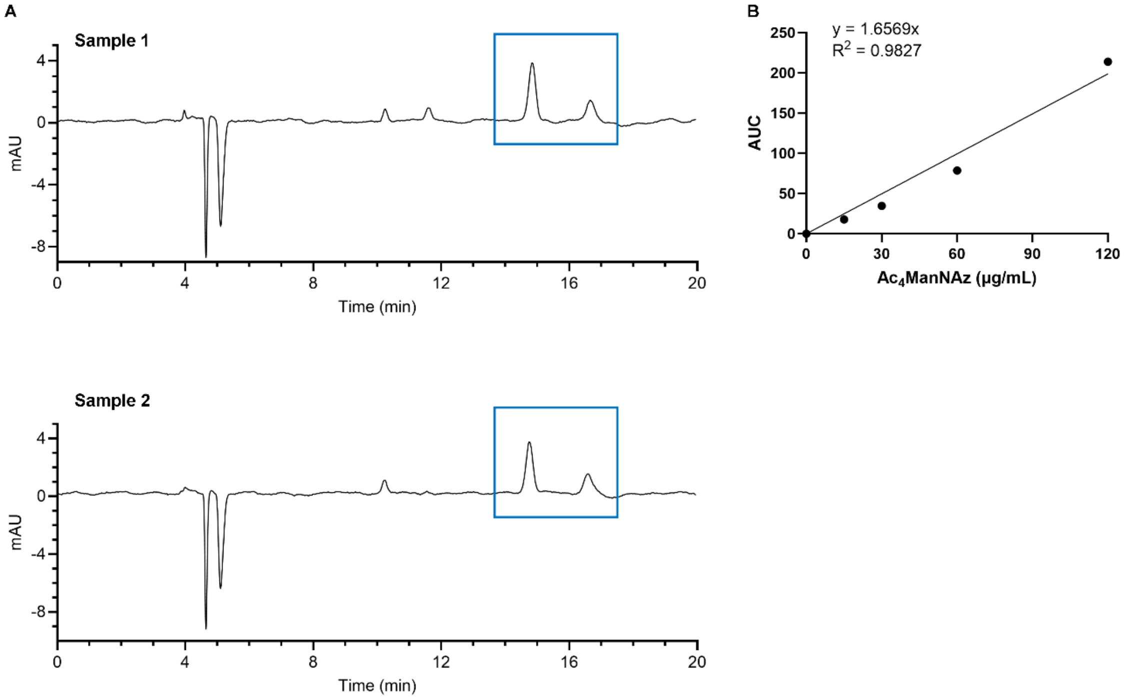

Supplemental Figure 2. Determination of Maz loading efficiency of MazNP via HPLC. (**A**) Representative HPLC chromatograms of Maz loaded MazNP for Sample 1 and Sample 2. The peak corresponding to Maz is highlighted in the blue square, showing at 15 – 16 minutes in both samples. (**B**) Standard curve of Maz, showing the integrated absorbance signal (area under the curve; AUC) at 220 nm plotted against Maz concentration (µg/mL). The loading efficiency (LE %) of Maz in MazNP was calculated as 6.3 ± 0.8%, defined as the loaded Maz/NP mass.

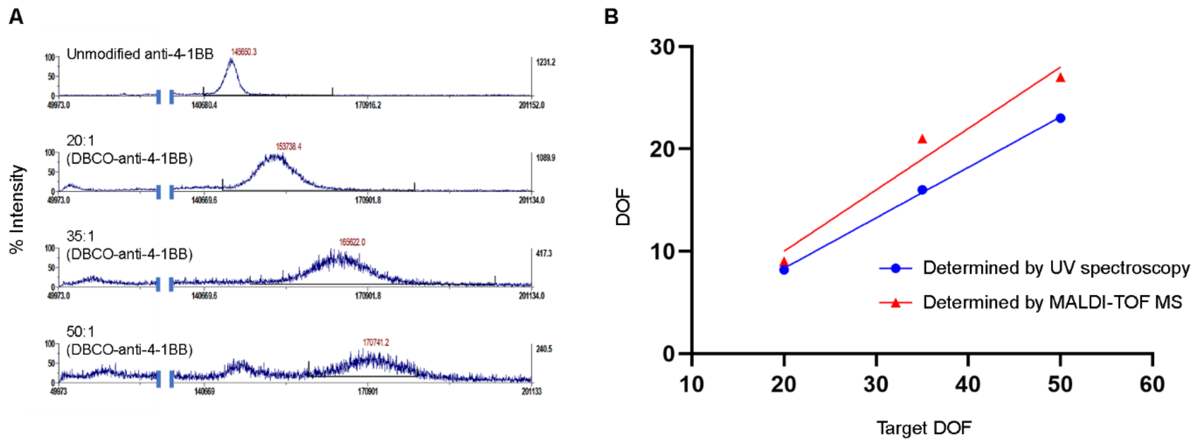

Supplemental Figure 3. Characterization of DBCO-functionalized anti-4-1BB. (**A**) MALDI-TOF mass spectra of unmodified anti-4-1BB and DBCO-functionalized anti-4-1BB with different molar ratios of DBCO to anti-4-1BB. (**B**) The DOF plot of different DBCO-functionalized anti-4-1BB determined by UV spectroscopic method and MALDI-TOF MS versus the molar equivalent of DBCO ligand used in anti-4-1BB functionalization (target DOF).

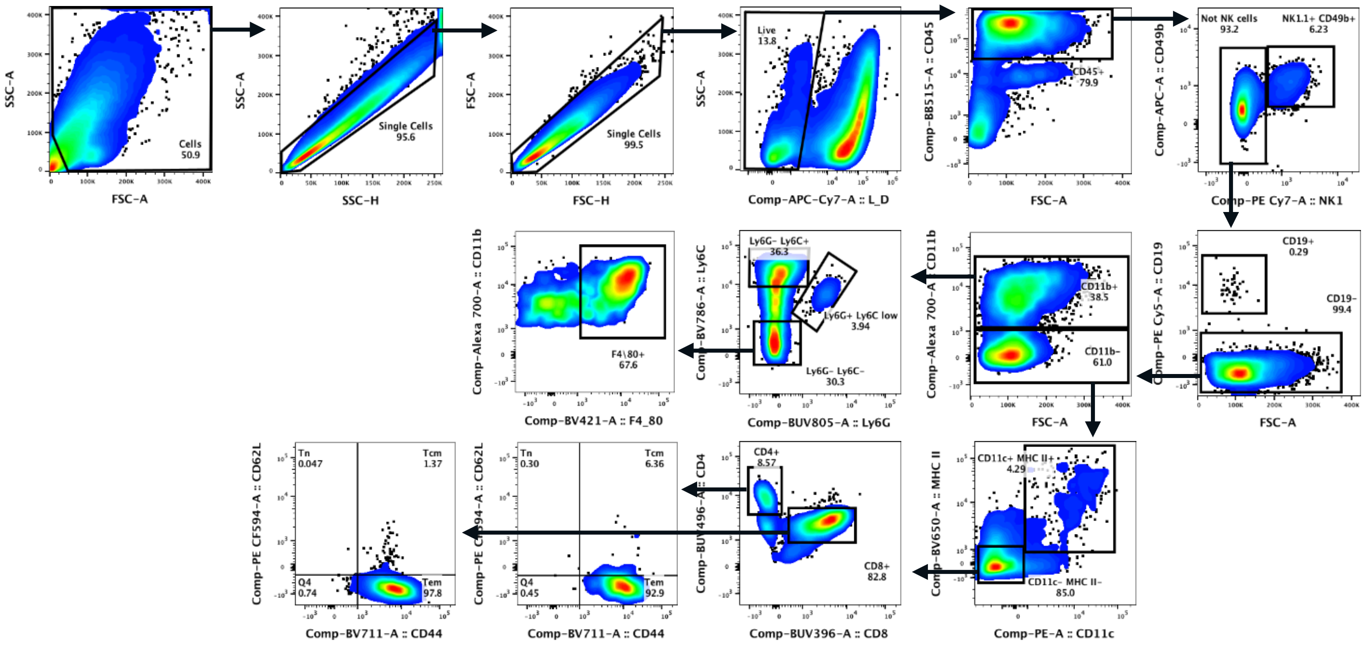

Supplemental Figure 4. Flow cytometry gating strategy for the analysis of immune cells *in vivo* in B16F10 tumor model. Populations of infiltrated NK cells (NK1.1^{+}CD49b^{+} in CD45^{+}), M-MDSCs (CD11b^{+}Ly6C^{+}), PMN-MDSCs (CD11b^{+}Ly6C^{lo}Ly6G^{+}), macrophages (CD11b^{+}F4/80^{+}), dendritic cells (DCs; CD11c^{+}MHCII^{+}), CD4+ T cells (CD4^{+}MHCII^{-} in CD45^{+}), CD4 Tem (CD62L^{-}CD44^{+} in CD4^{+} T cells), CD8^{+} T cells (CD8^{+}MHCII^{-} in CD45^{+}), Tem (CD62L^{-}CD44^{+} in CD8^{+} T cells), and Tcm (CD62L^{+}CD44^{+} in CD8^{+} T cells). The gating strategy for all samples was set to remove large clumps or aggregates of cells (SSC-A and SSC-H gating; FSC-A and FSC-H gating), cell debris, and dead cells (Zombie NIR gating).

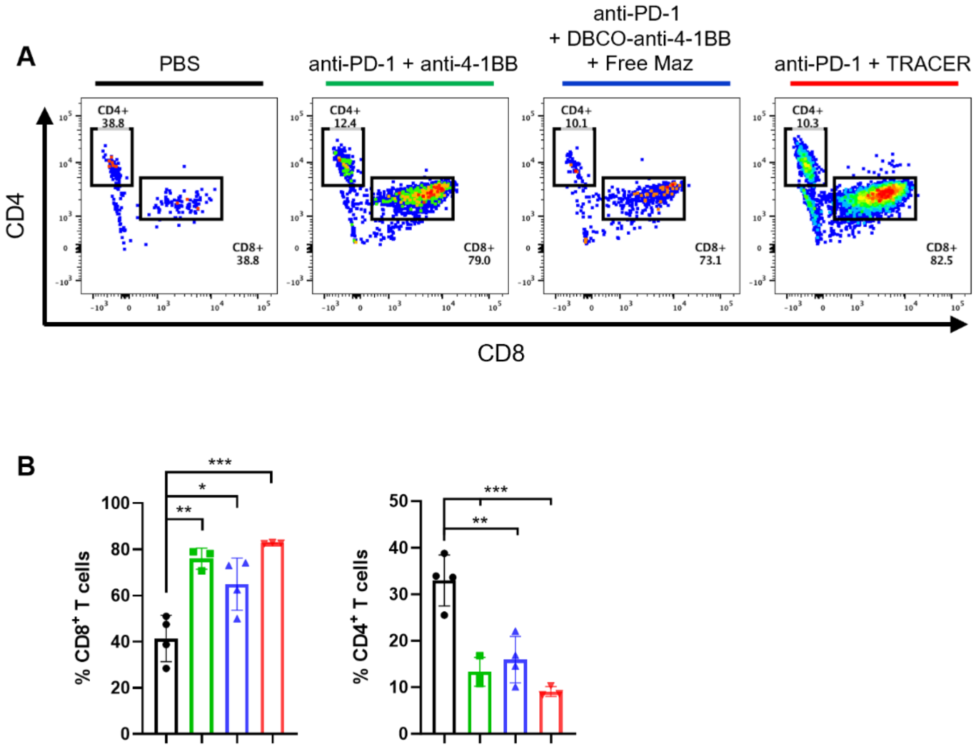

Supplemental Figure 5. Representative flow cytometry dot plots (A) and percentage of CD8$^+$ T and CD4$^+$ T cells (B). *: $p < 0.05$, **: $p < 0.01$, and ***: $p < 0.001$ by Tukey's multiple comparisons test following one-way ANOVA.

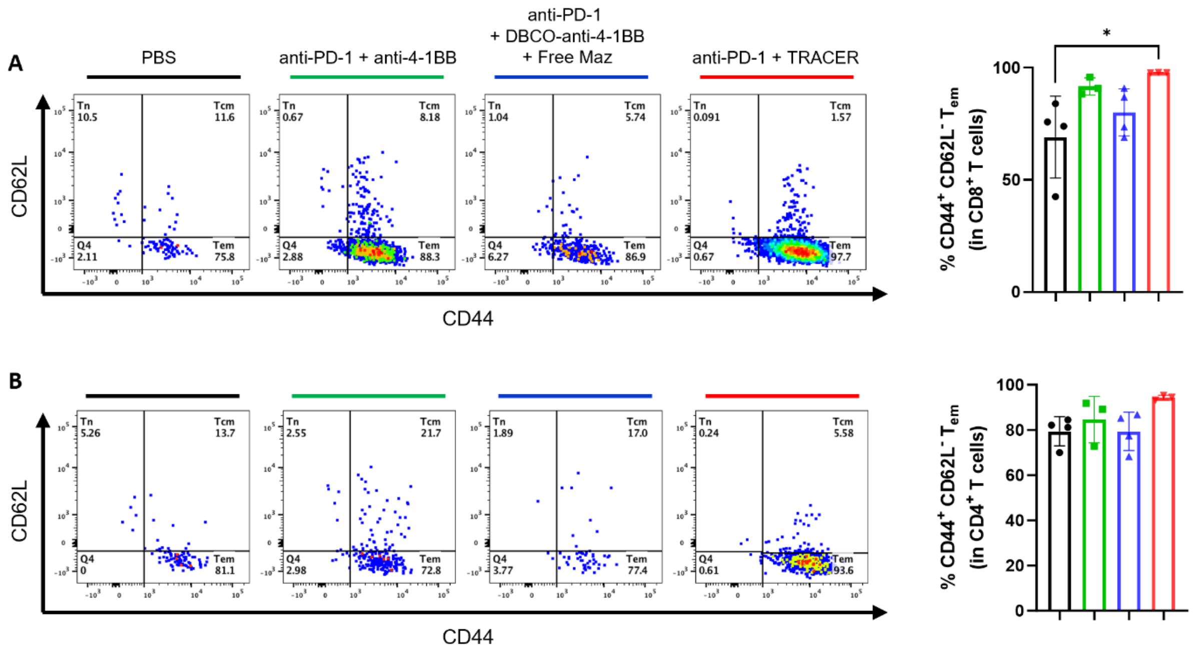

Supplemental Figure 6. Representative images of flow cytometry dot plots and percentage of Tem ($CD44^+CD62^-$) in $CD8^+$ (A) and in $CD4^+$ (B). *: $p < 0.05$ by Tukey's multiple comparisons test following one-way ANOVA.

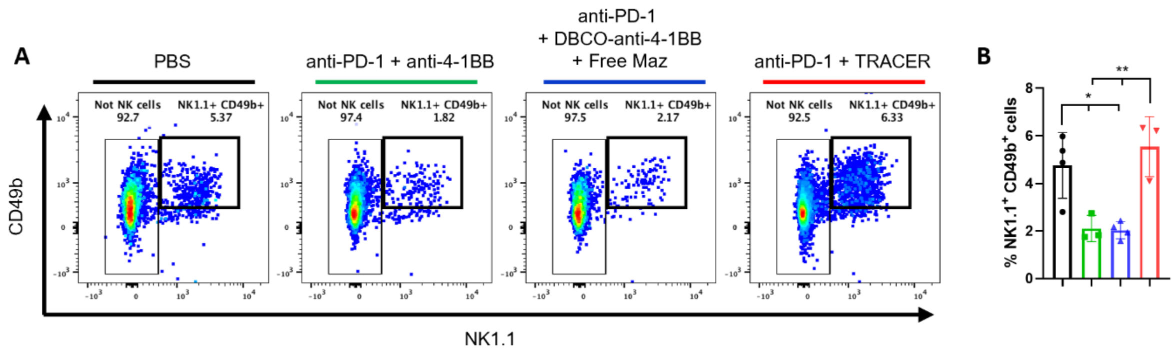

Supplemental Figure 7. Representative flow cytometry dot plots (A) and percentage of NK1.1$^+$CD49b$^+$ cells in tumors (B). *: $p < 0.05$ and **: $p < 0.01$ by Tukey's multiple comparisons test following one-way ANOVA.

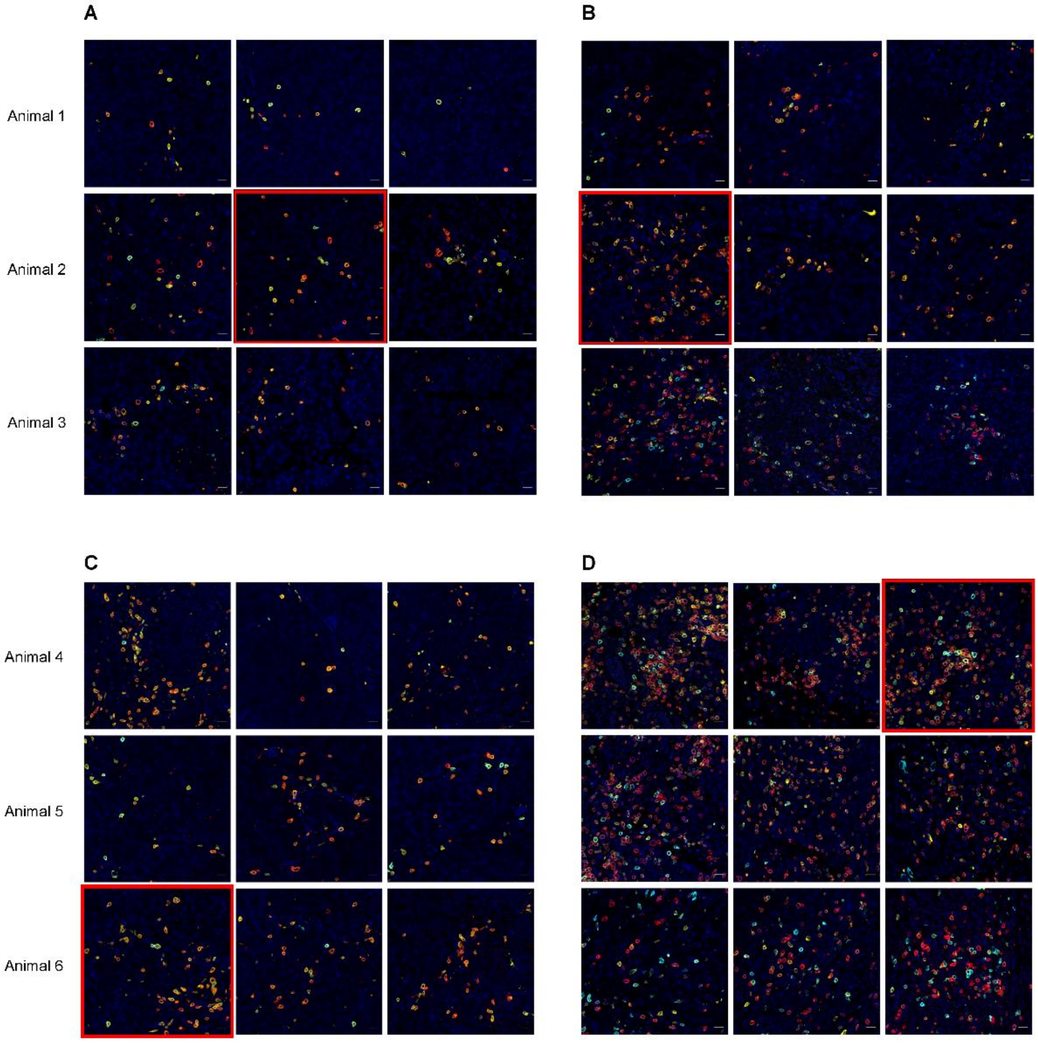

Supplemental Figure 8. All immunofluorescence microscopy of tumor sections from B16F10 melanoma tumor model. (**A**) PBS, (**B**) anti-PD-1+anti-4-1BB, (**C**) anti-PD-1+DBCO-anti-4-1BB+Free Maz, and (**D**) anti-PD-1+TRACER. Images were collected from three randomly selected fields per slide (Yellow: CD3; Cyan: CD4; Red: CD8; Blue: nuclei stained with Hoechst 33342). Mice (*n* = 3 per group) were given the same treatment as the antitumor efficacy study and sacrificed 5 days after the last treatment. Scale bars = 20 µm. Images used in the main text (Figure 3E) are highlighted with a red outline.

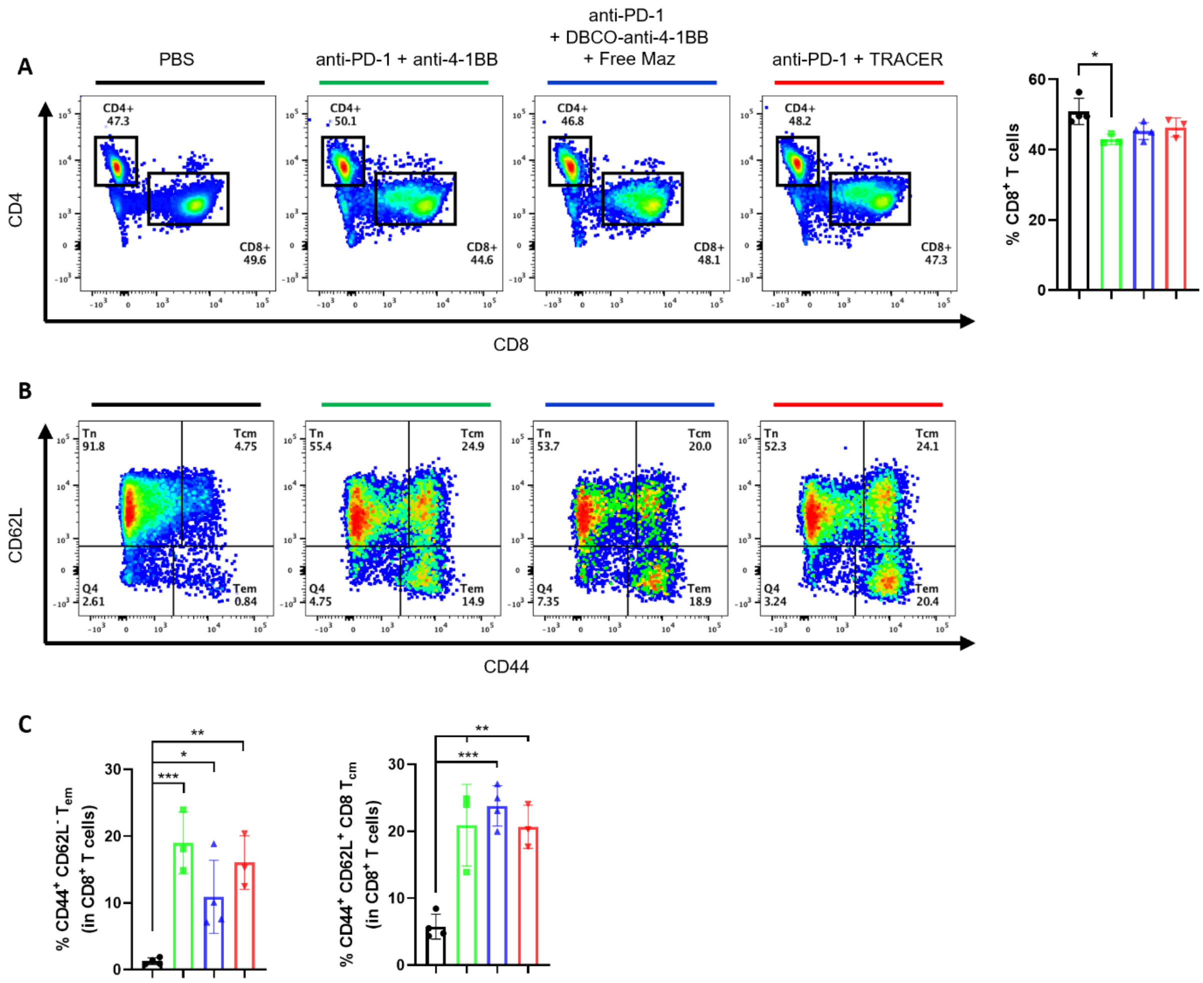

Supplemental Figure 9. Representative flow cytometry dot plots and percentage of CD8$^+$ T cells (A), and Tem (CD44$^+$CD62$^-$) and Tcm (CD44$^+$CD62$^+$) in CD8$^+$ (B and C) in TDLN. *: $p < 0.05$, **: $p < 0.01$, and ***: $p < 0.001$ by Tukey's multiple comparisons test following one-way ANOVA.

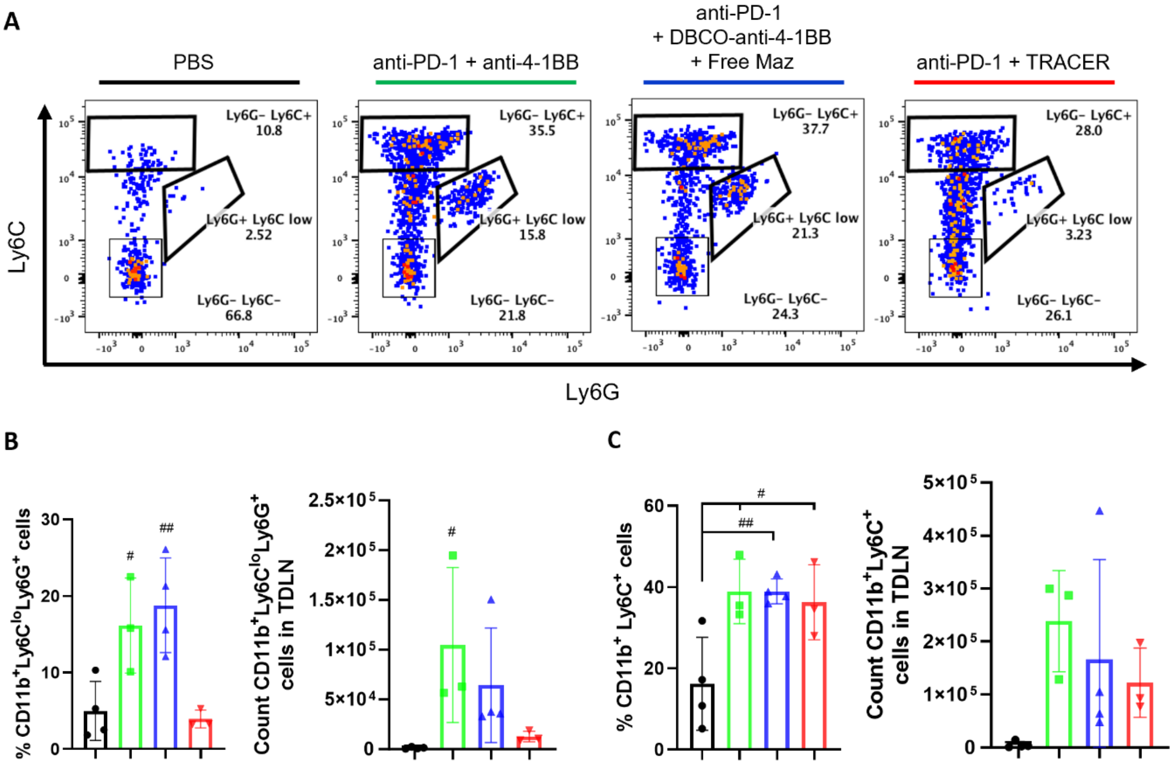

Supplemental Figure 10. (A) Representative flow cytometry dot plots and quantification of (B) PMN-MDSCs (CD11b^{+}Ly6C^{lo}Ly6G^{+}) and (C) M-MDSCs (CD11b^{+}Ly6C^{+}) in TDLNs. #: $p < 0.05$ and ##: $p < 0.01$ vs. PBS by Dunnett's multiple comparisons test following one-way ANOVA. *: $p < 0.05$ by Tukey's multiple comparisons test following one-way ANOVA.

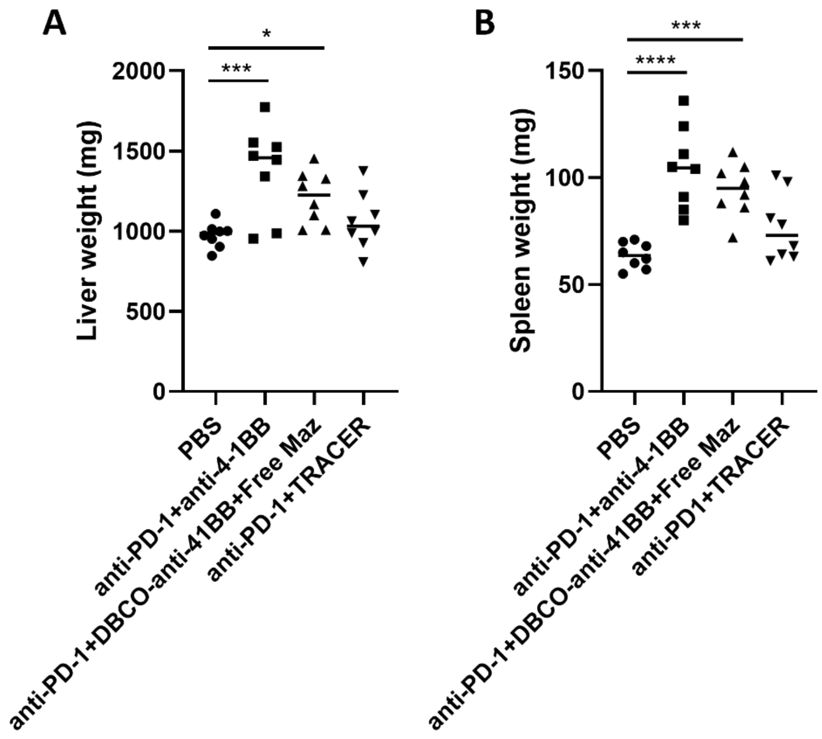

Supplemental Figure 11. Liver (A) and spleen (B) weights from B16F10 tumor-bearing mice (*n* = 8 per group) treated with PBS, anti-PD-1+anti-4-1BB, anti-PD-1+DBCO-anti-4-1BB+free Maz, or anti-PD-1+TRACER. *: $p < 0.05$; ***: $p < 0.001$; ****: $p < 0.0001$ by Dunnett's multiple comparisons test following one-way ANOVA.

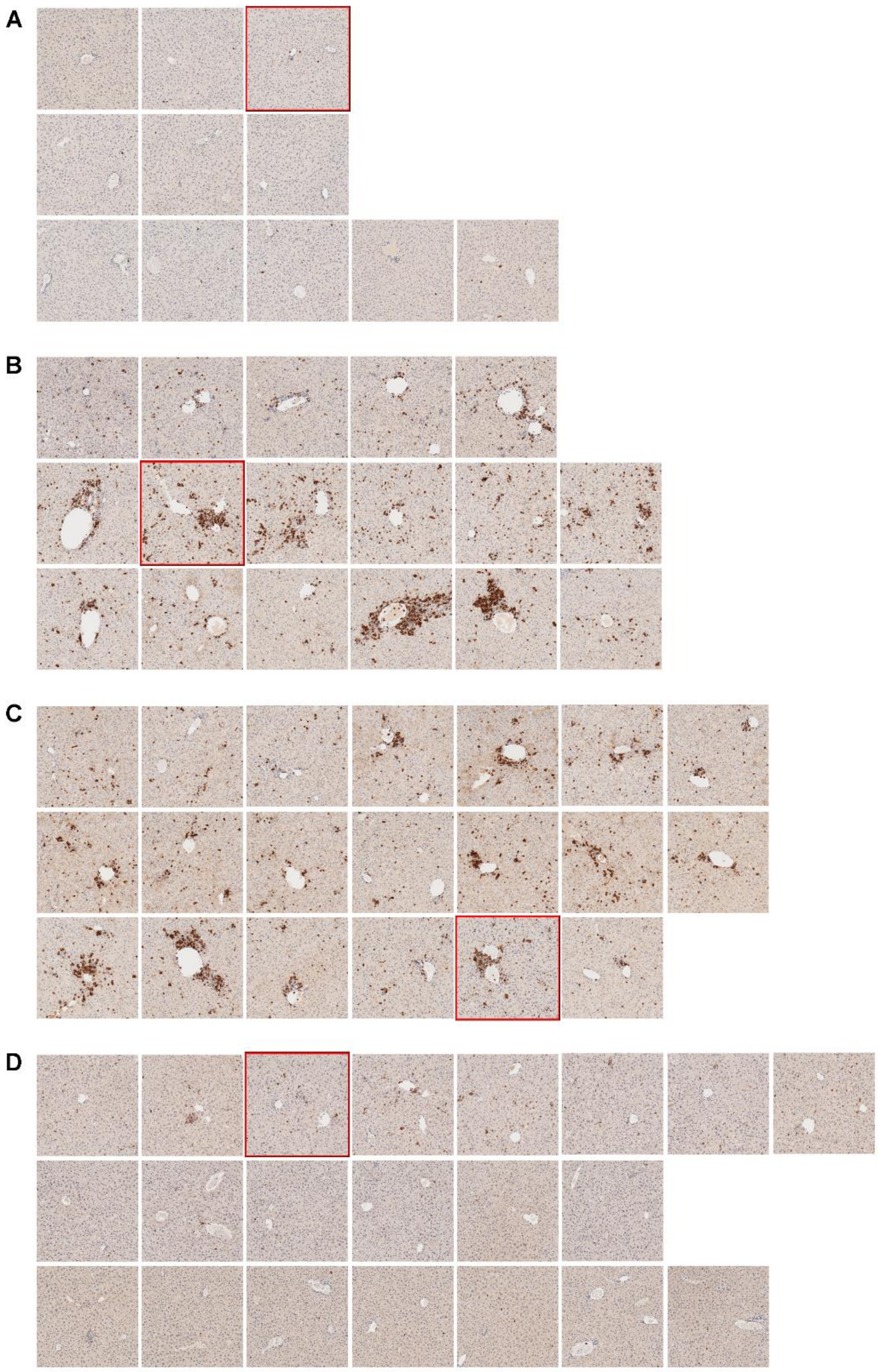

Supplemental Figure 12. All images of liver sections stained against CD8. (**A**) PBS (11 images), (**B**) anti-PD-1+anti-4-1BB (17 images), (**C**) anti-PD-1+DBCO-anti-4-1BB+free Maz (20 images), and (**D**) anti-PD-1+TRACER (21 images). Images were collected from randomly selected fields in the slide. Mice (*n* = 3 per group) were given the same treatment as the antitumor efficacy study and sacrificed 5 days after the last treatment. Scale bars = 50 µm. Images used in the main text (Figure 5C) are highlighted with a red outline.

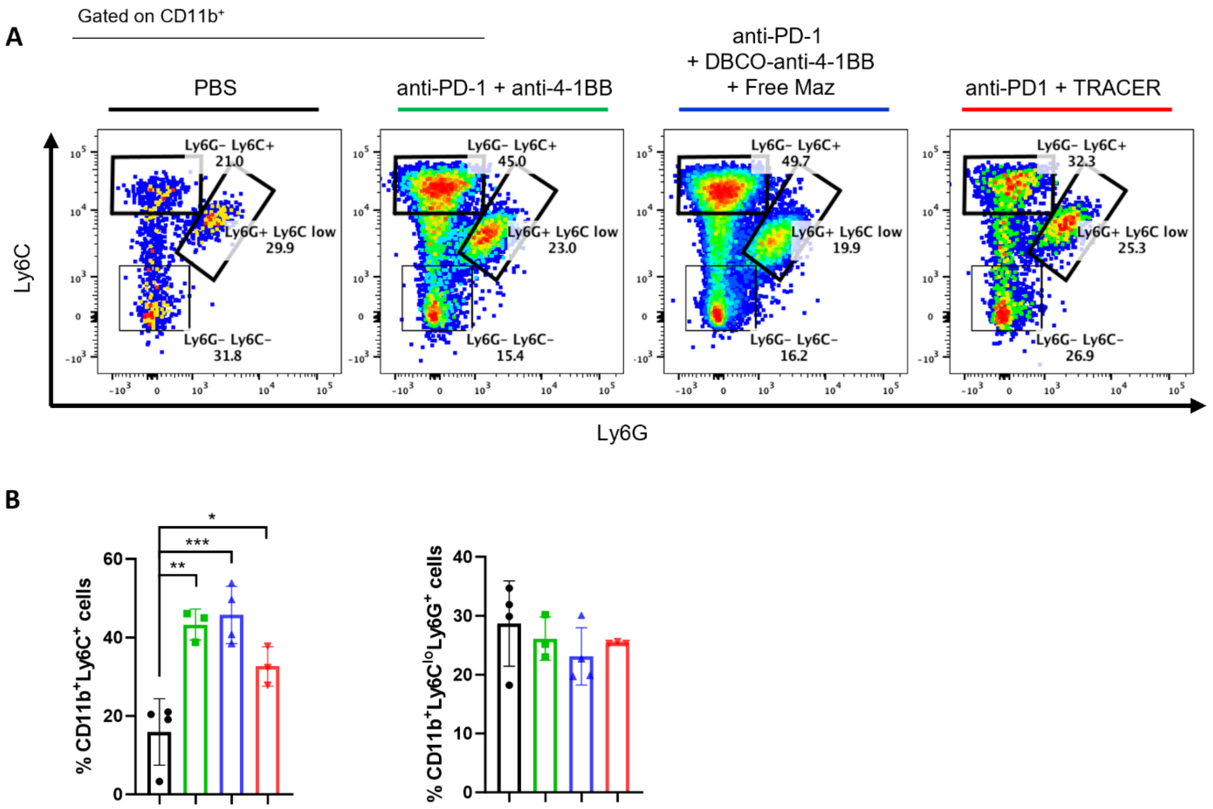

Supplemental Figure 13. Representative flow cytometry dot plots (A) and percentage of M-MDSCs (CD11b$^+$Ly6C$^+$) and PMN-MDSCs (CD11b$^+$Ly6C^{lo}Ly6G$^+$) (B) in livers. *: $p < 0.05$; **: $p < 0.01$; ***: $p < 0.001$ by Tukey's multiple comparisons test following one-way ANOVA.

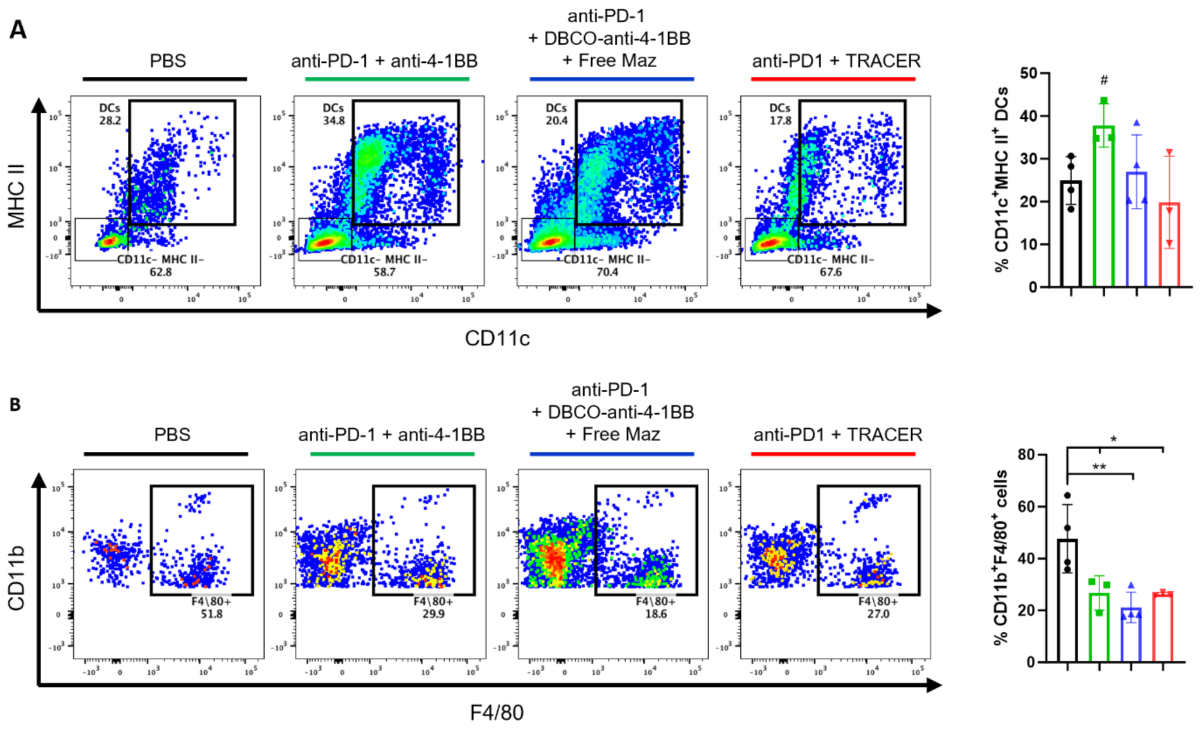

Supplemental Figure 14. Representative flow cytometry dot plots and percentage of DCs (CD11c$^+$MHCII$^+$) (A) and macrophages (CD11b$^+$F4/80$^+$) (B) in livers. #: p < 0.05 by Dunnett's multiple comparisons test following one-way ANOVA, and *: p < 0.05; **: p < 0.01 by Tukey's multiple comparisons test following one-way ANOVA.

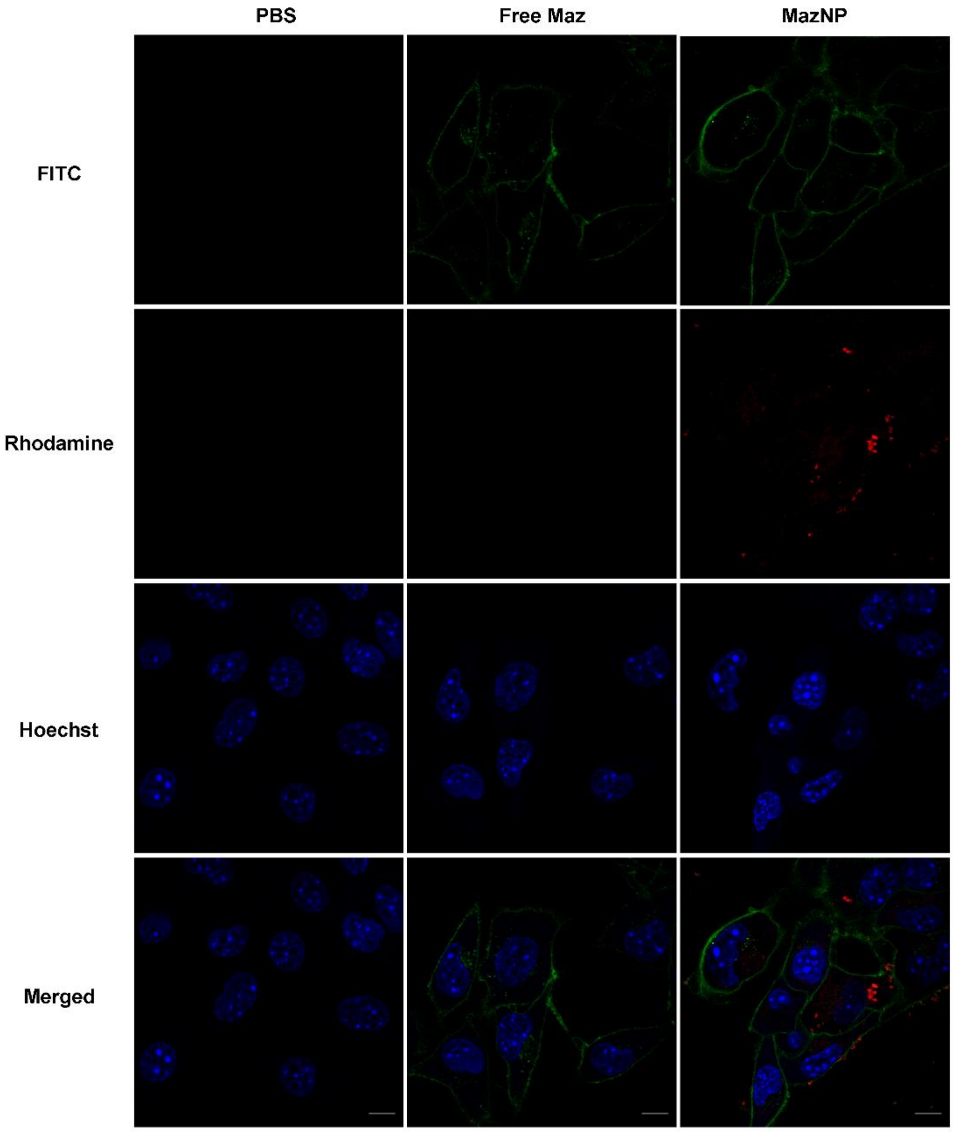

Supplemental Figure 15. Azide group generation on the B16F10 cell surface after 6 h incubation with PBS, free Maz or non-PEGylated MazNP. The cells were imaged with confocal microscopy (Green: streptavidin-FITC; Red: rhodamine-labeled MazNP; Blue: nuclei stained with Hoechst 33342). Scale bars: 10 μm.

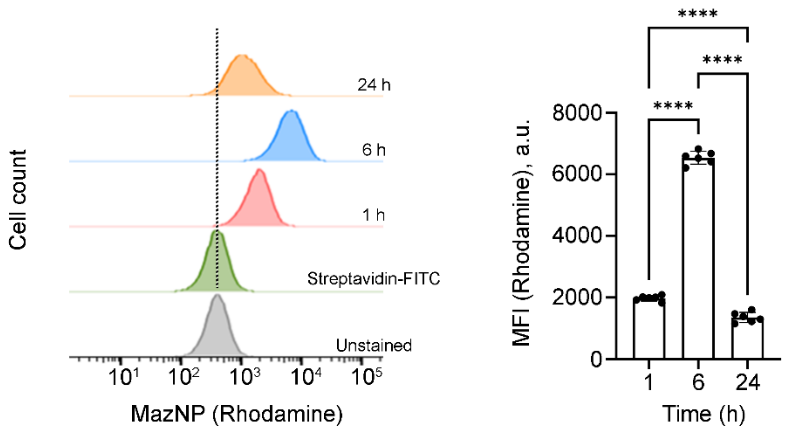

Supplemental Figure 16. Time-dependent cellular uptake of rhodamine-labeled MazNPs in J774a.1 macrophages. Representative flow cytometry histogram showing rhodamine signal at 1 h, 6 h, and 24 h post-treatment. Quantification of cellular rhodamine intensity (MFI). ****: $p < 0.0001$ by Tukey's multiple comparisons test following one-way ANOVA.

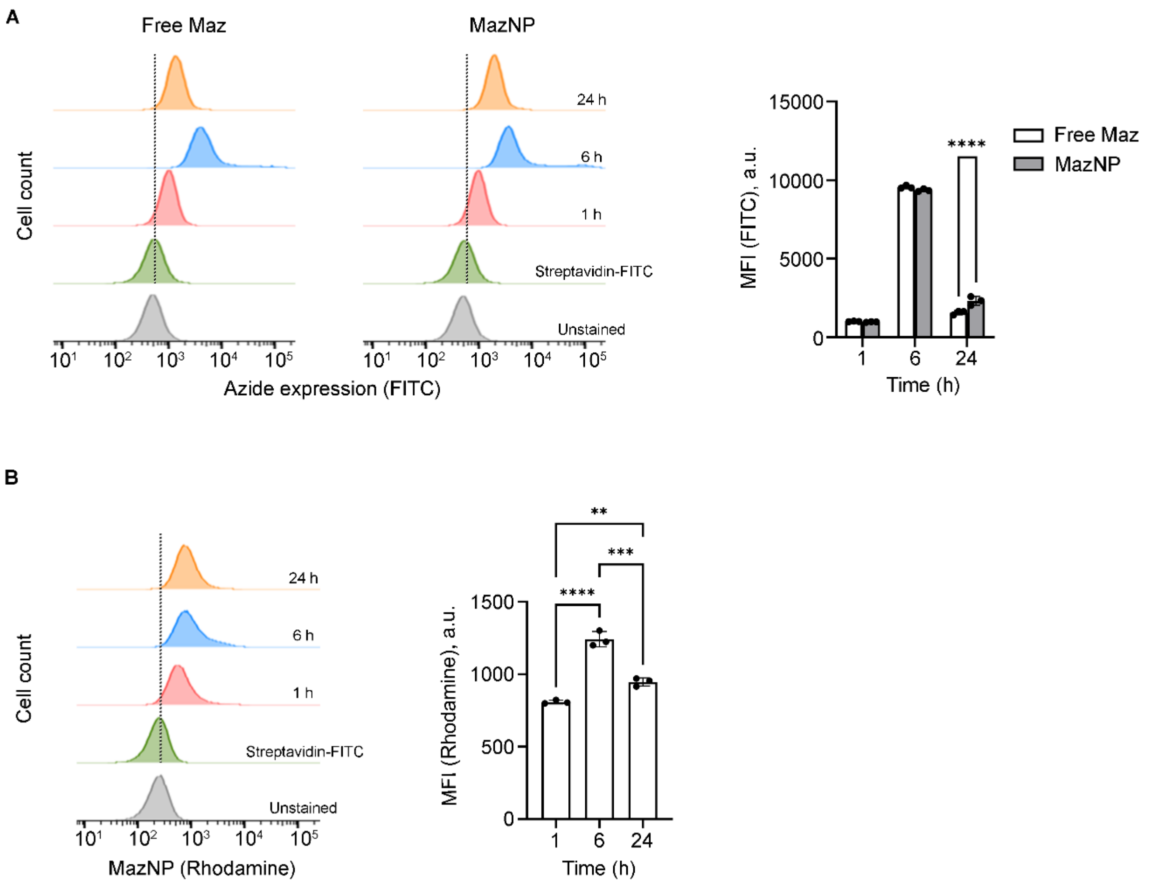

Supplemental Figure 17. Time-dependent analysis of MazNP processing in B16F10 cells. (**A**) Representative flow cytometry histograms showing cell-surface azide expression in free Maz and MazNP treated cells at 1 h, 6 h, and 24 h. Quantification of cell-surface azide expression (MFI, streptavidin-FITC). ****: $p < 0.0001$ by by Sidak's multiple comparisons test following two-way ANOVA. (**B**) Representative flow cytometry histogram showing rhodamine signal at 1 h, 6 h, and 24 h post-treatment. Quantification of cellular rhodamine intensity (MFI). **: $p < 0.01$; ***: $p < 0.001$ and ****: $p < 0.0001$ by Tukey's multiple comparisons test following one-way ANOVA.

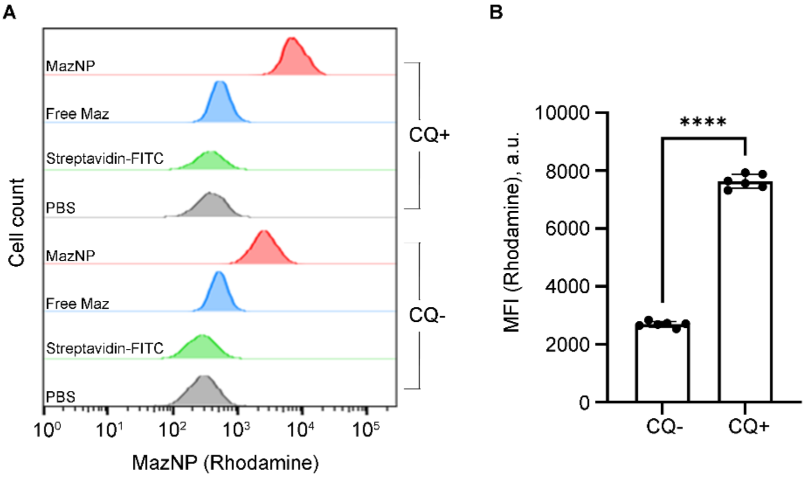

Supplemental Figure 18. Effect of chloroquine on MazNP processing in J774a.1 macrophages. (**A**) Representative of flow cytometry histogram showing rhodamine signal in cells with CQ (CQ^+) or without CQ (CQ^-) pretreatment. (**B**) Quantification of rhodamine intensity (MFI). ****: $p < 0.0001$ by unpaired two-tailed *t*-test.

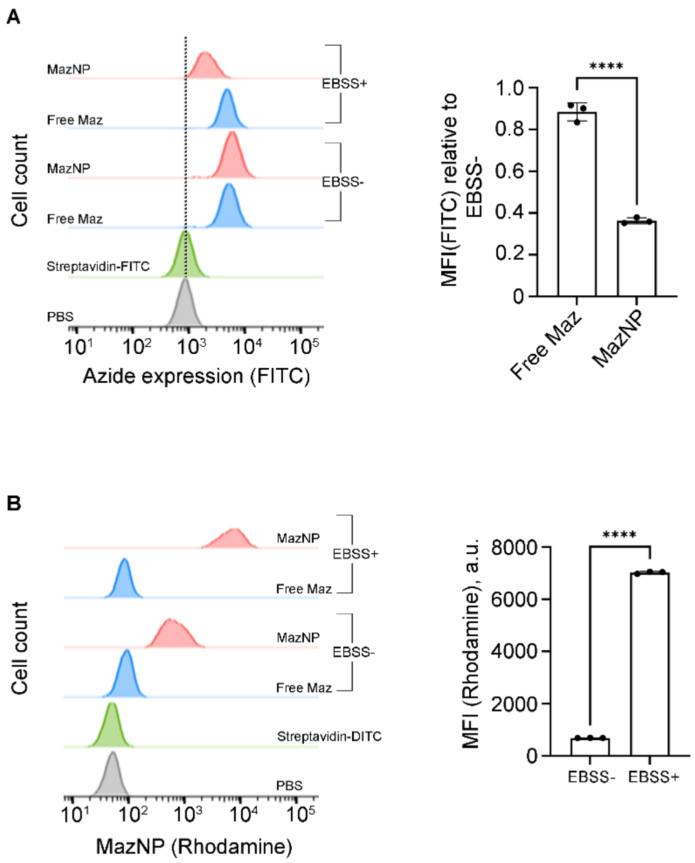

Supplemental Figure 19. Effect of EBSS on MazNP processing in B16F10 cells. (A) Representative of flow cytometry histogram showing cell-surface azide expression in cells treated with free Maz and MazNP. Quantification of cell-surface azide expression is presented as the ratio of MFI of streptavidin-FITC in EBSS-incubated cells relative non-incubated cells (EBSS+ to EBSS-). ****: $p < 0.0001$ by unpaired two-tailed t-test. (**B**) Representative of flow cytometry histogram showing rhodamine signal in cells incubated with or without EBSS. Quantification of rhodamine MFI. ****: $p < 0.0001$ by unpaired two-tailed *t*-test.

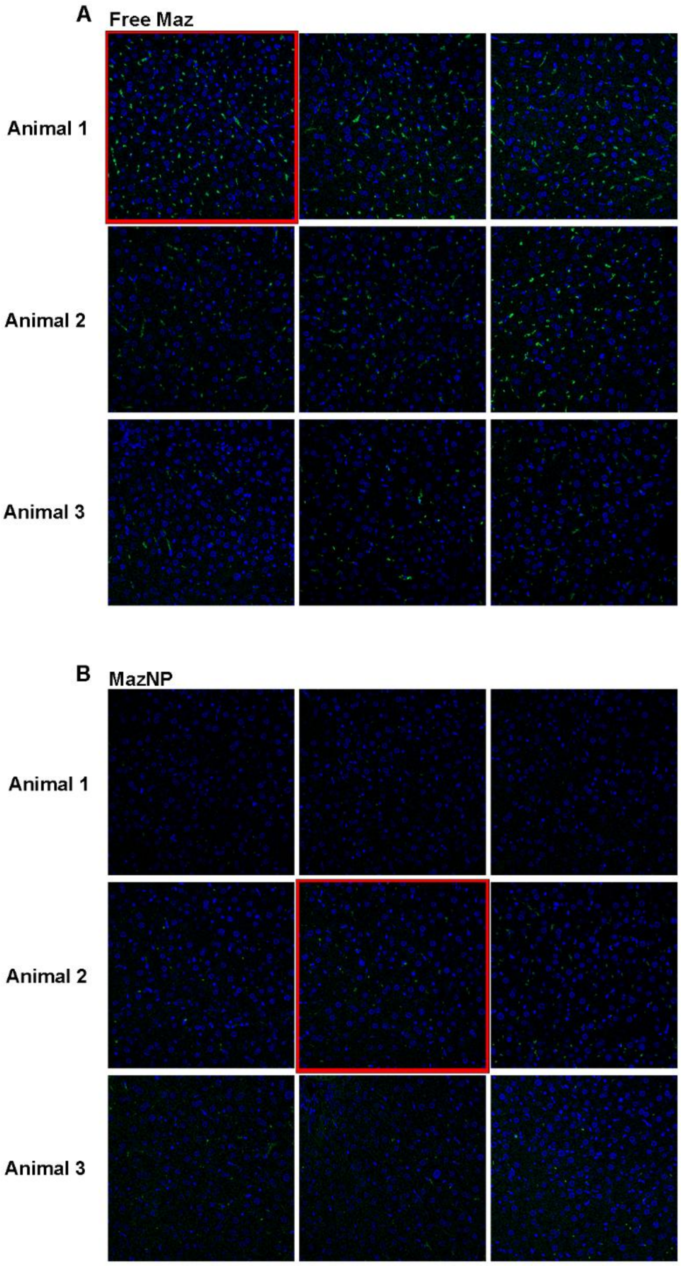

Supplemental Figure 20. *In vivo* azide labeling in livers. All fluorescence images of liver tissue sections from B16F10 tumor-bearing mice treated with free Maz (A) or MazNP (B). B16F10 tumor-bearing mice (*n* = 3 per group) were injected IV once daily for two days of free Maz or MazNP (eq. to Maz 35 mg/kg in total). After 24 h, livers were harvested, and azide groups expressed in liver were labeled with DBCO-Cy5 (green) for 1 h. Cell nuclei were stained with Hoechst 33258 (Blue). Scale bars: 20 µm. Images used in the main text (Figure 7B) are highlighted with a red outline.

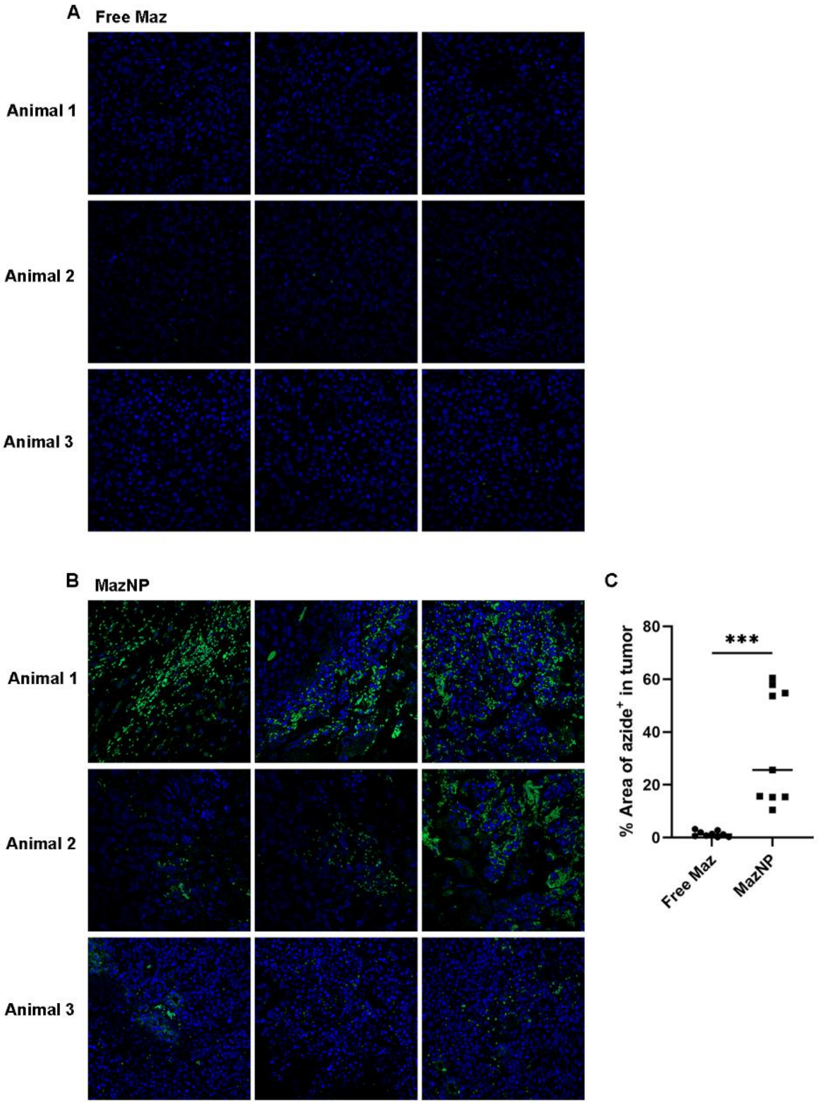

Supplemental Figure 21. ***In vivo*** **azide labeling in tumors. All fluorescence images of liver tissue sections from B16F10 tumor-bearing mice treated with free Maz (A) or MazNP (B).** B16F10 tumor-bearing mice (n = 3 per group) were injected IV once daily for two days with either free Maz or MazNP (eq. to Maz 35 mg/kg in total). After 24 h, tumors were harvested, and azide groups expressed in tumor were labeled with DBCO-Cy5 (green) for 1 h. Cell nuclei were stained with Hoechst 33258 (Blue). Scale bars: 20 µm. (**C**) Quantitative analysis of streptavidin-FITC-stained tumor. % Area of azide groups expressed in tumor was estimated as the area of streptavidin-FITC (green) divided by the tissue in the field of view area (outlined by blue Hoechst 33258 staining). ***: $p < 0.001$ by unpaired two-tailed t-test.

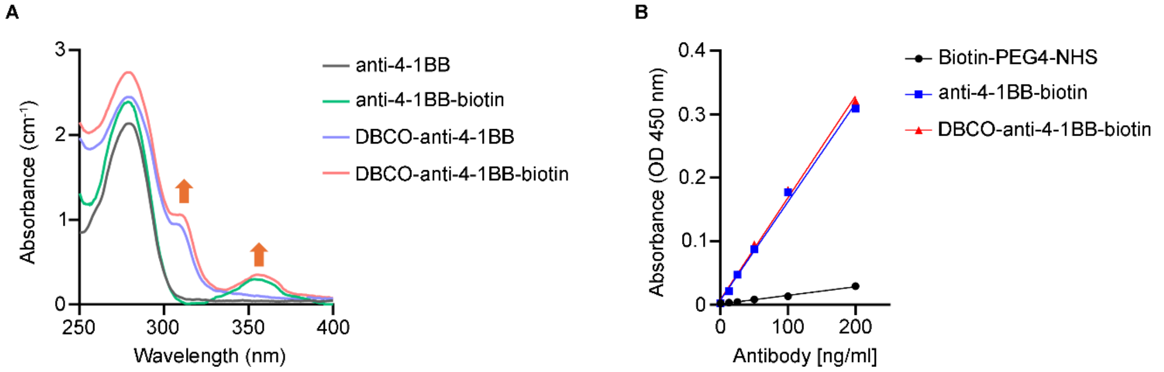

Supplemental Figure 22. Characterization of biotin-conjugated anti-4-1BB (anti-4-1BB-biotin) and DBCO-anti-4-1BB (DBCO-anti-4-1BB-biotin). Antibodies were functionalized with DBCO-PEG$_{13}$-NHS via an amine-NHS ester coupling reaction and subsequently conjugated to biotin-PEG4-NHS ester at a molar ratio of 1.3:1, producing DBCO-anti-4-1BB-biotin. (**A**) UV-visible absorption spectra of anti-4-1BB-biotin and DBCO-anti-4-1BB-biotin at a concentration of 1 mg/mL. The UV absorption bands at 310 nm and 354 nm correspond to the absorbance from the conjugated DBCO group and biotin, respectively (arrows). (**B**) Absorbance at 450 nm (OD450) for varying concentrations of the antibody, measured by UV-visible spectroscopy. ELISA validation confirmed that biotin-labeled anti-4-1BB and DBCO-anti-4-1BB antibodies exhibited a linear response in absorbance with increasing concentration, verifying their suitability for quantification.

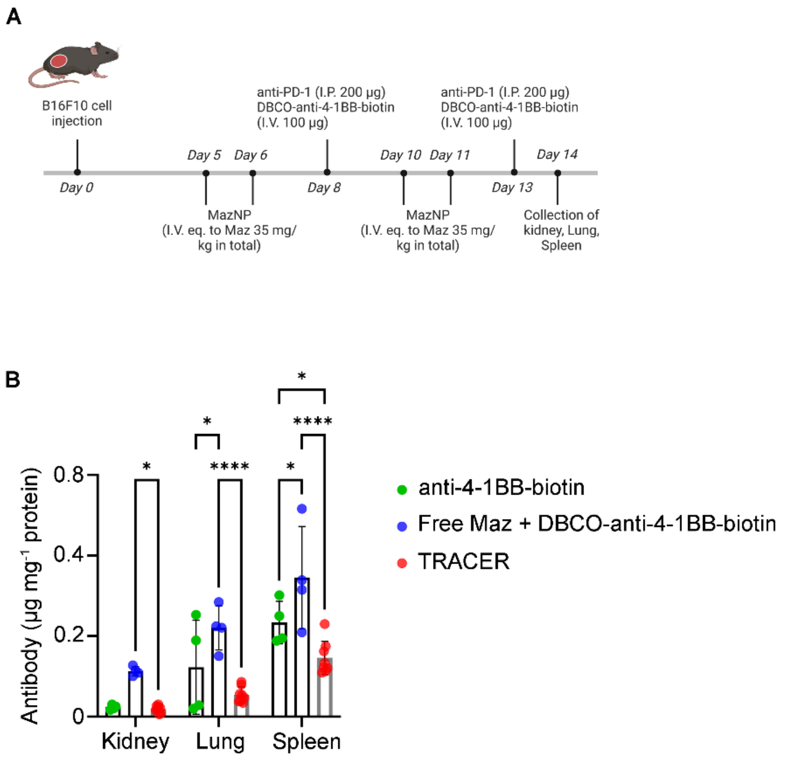

Supplemental Figure 23. Tissue Distribution of anti-4-1BB-biotin and DBCO-anti-4-1BB-biotin. (**A**) Schematic of B16F10 tumor inoculation, treatment regimen, and time point for collection of tissues (n = 4). (**B**) Biotin ELISA quantification of anti-4-1BB-biotin and DBCO-anti-4-1BB-biotin in the kidney, lung, and spleen. Data are presented as antibody concentration relative to protein content (µg antibody per mg protein). *: $p < 0.05$ and ****: $p < 0.0001$ by Tukey's multiple comparisons test following two-way ANOVA.

Supplemental References

1. Malvern Instruments Limited. Dynamic light scattering – Common terms defined (MRK 1764-01). https://www.malvernpanalytical.com/en/learn/knowledge-center/Whitepapers/WP111214DLSTermsDefined. Accessed June, 2024.
2. International Organization for Standardization. ISO 22412:2008(en) Particle size analysis - Dynamic light scattering (DLS). https://www.iso.org/obp/ui/#iso:std:iso:22412:ed-1:v1:en. Accessed June, 2024.
3. Au KM, Park SI, and Wang AZ. Trispecific natural killer cell nanoengagers for targeted chemoimmunotherapy. *Sci Adv.* 2020;6(27):eaba8564.
4. Au KM, Tripathy A, Lin CP, Wagner K, Hong S, Wang AZ, et al. Bespoke Pretargeted Nanoradioimmunotherapy for the Treatment of Non-Hodgkin Lymphoma. *ACS Nano.* 2018;12(2):1544-63.
5. Quiles S, Raisch KP, Sanford LL, Bonner JA, and Safavy A. Synthesis and preliminary biological evaluation of high-drug-load paclitaxel-antibody conjugates for tumor-targeted chemotherapy. *J Med Chem.* 2010;53(2):586-94.
6. Compte M, Harwood SL, Munoz IG, Navarro R, Zonca M, Perez-Chacon G, et al. A tumor-targeted trimeric 4-1BB-agonistic antibody induces potent anti-tumor immunity without systemic toxicity. *Nat Commun.* 2018;9(1):4809.
7. Tomayko MM, and Reynolds CP. Determination of subcutaneous tumor size in athymic (nude) mice. *Cancer Chemother Pharmacol.* 1989;24(3):148-54.
8. Kwong B, Gai SA, Elkhader J, Wittrup KD, and Irvine DJ. Localized immunotherapy via liposome-anchored Anti-CD137 + IL-2 prevents lethal toxicity and elicits local and systemic antitumor immunity. *Cancer Res.* 2013;73(5):1547-58.
